# Transcriptomic and proteomic profiles of fetal versus adult mesenchymal stromal cells and mesenchymal stromal cell-derived extracellular vesicles

**DOI:** 10.1186/s13287-024-03683-7

**Published:** 2024-03-13

**Authors:** Emine Begüm Gençer, Yuk Kit Lor, Fawaz Abomaray, Samir El Andaloussi, Maria Pernemalm, Nidhi Sharma, Daniel W Hagey, André Görgens, Manuela O Gustafsson, Katarina Le Blanc, Mawaddah Asad Toonsi, Lilian Walther-Jallow, Cecilia Götherström

**Affiliations:** 1 Division of Obstetrics and Gynecology, Department of Clinical Science, Intervention and Technology, Karolinska Institutet, Stockholm, Sweden; 2 Biomolecular Medicine, Clinical Research Center, Department of Laboratory Medicine, Karolinska Institutet, Stockholm, Sweden; 3 Department of Cellular Therapy and Allogeneic Stem Cell Transplantation (CAST), Karolinska University Hospital Huddinge and Karolinska Comprehensive Cancer Center, Stockholm, Sweden; 4 Cancer Proteomics Mass Spectrometry, Science for Life Laboratory, Department of Oncology- Pathology, Karolinska Institutet, Stockholm, Sweden; 5 Institute for Transfusion Medicine, University Hospital Essen, University of Duisburg-Essen, Essen, Germany; 6 Division of Clinical Immunology and Transfusion Medicine, Karolinska Institutet, Stockholm, Sweden; 7 Department of Pediatrics, College of Medicine, Umm Al-Qura University, Makkah, Saudi Arabia

**Keywords:** Mesenchymal stromal cells, Fetal mesenchymal stem cells, Biosignature, Transcriptomic, Proteomic, Extracellular vesicles

## Abstract

**Background:**

Mesenchymal stem/stromal cells (MSCs) can regenerate tissues through engraftment and differentiation but also via paracrine signalling via extracellular vesicles (EVs). Fetal-derived MSCs (fMSCs) have been shown, both in vitro and in animal studies, to be more efficient than adult MSC (aMSCs) in generating bone and muscle but the underlying reason for this difference has not yet been clearly elucidated. In this study, we aimed to systematically investigate the differences between fetal and adult MSCs and MSC-derived EVs at the phenotypic, RNA, and protein levels.

**Methods:**

We carried out a detailed and comparative characterization of culture-expanded fetal liver derived MSCs (fMSCs) and adult bone marrow derived MSCs (aMSCs) phenotypically, and the MSCs and MSC-derived EVs were analysed using transcriptomics and proteomics approaches with RNA Sequencing and Mass Spectrometry.

**Results:**

Fetal MSCs were smaller, exhibited increased proliferation and colony-forming capacity, delayed onset of senescence, and demonstrated superior osteoblast differentiation capability compared to their adult counterparts. Gene Ontology analysis revealed that fMSCs displayed upregulated gene sets such as “Positive regulation of stem cell populations”, “Maintenance of stemness” and “Muscle cell development/contraction/Myogenesis” in comparison to aMSCs. Conversely, aMSCs displayed upregulated gene sets such as “Complement cascade”, “Adipogenesis”, “Extracellular matrix glycoproteins” and “Cellular metabolism”, and on the protein level, “Epithelial cell differentiation” pathways. Signalling entropy analysis suggested that fMSCs exhibit higher signalling promiscuity and hence, higher potency than aMSCs. Gene ontology comparisons revealed that fetal MSC-derived EVs (fEVs) were enriched for “Collagen fibril organization”, “Protein folding”, and “Response to transforming growth factor beta” compared to adult MSC-derived EVs (aEVs), whereas no significant difference in protein expression in aEVs compared to fEVs could be detected.

**Conclusions:**

This study provides detailed and systematic insight into the differences between fMSCs and aMSCs, and MSC-derived EVs. The key finding across phenotypic, transcriptomic and proteomic levels is that fMSCs exhibit higher potency than aMSCs, meaning they are in a more undifferentiated state. Additionally, fMSCs and fMSC-derived EVs may possess greater bone forming capacity compared to aMSCs. Therefore, using fMSCs may lead to better treatment efficacy, especially in musculoskeletal diseases.

**Supplementary Information:**

The online version contains supplementary material available at 10.1186/s13287-024-03683-7.

## Graphical abstract

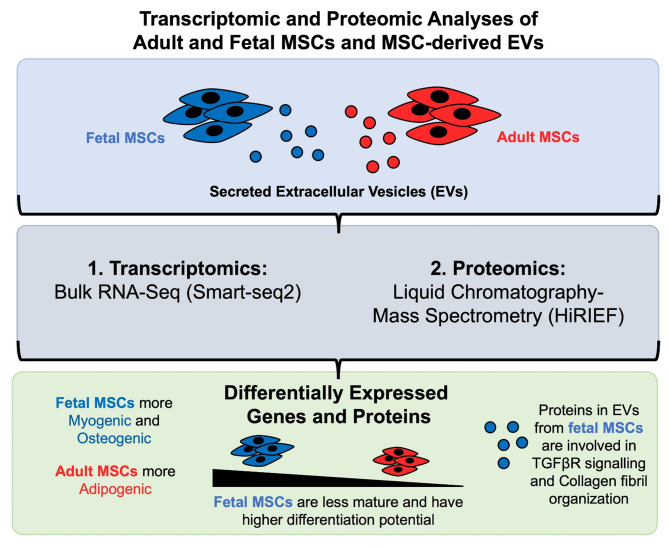

## Background

Mesenchymal stem/stromal cells (MSCs) are promising candidates in regenerative medicine due to their contribution to osteogenic, chondrogenic, and myogenic lineages. MSCs have minimal oncogenic risk, exhibit an immune-evasive phenotype allowing allogenic transplantations without immunosuppression and can be easily manufactured at large scale. The mechanism behind the therapeutic effect of MSCs was initiallybelieved to be mainly through engraftment in host tissue and replacement of depleted or faulty cell types via differentiation. However, recent studies have indicated that MSCs may also exhibit additional beneficial effects through paracrine pathways, involving the generation and release of soluble factors and extracellular vesicles (EVs) mediating immune-modulatory and trophic functions [1–3]. Hence, apart from their ability to differentiate into various lineages, MSCs may promote tissue repair in animal models through paracrine signalling [4–6]. Due to these characteristics, MSCs, predominantly from adult sources (aMSCs) such as bone marrow, have been investigated in clinical trials for variety of disorders [7, 8]. However, fetal MSCs offer higher therapeutic potential compared to MSCs derived from adult sources [9, 10]. Fetal MSCs are currently being investigated as a treatment for Osteogenesis Imperfecta (OI), a rare genetic disorder characterised by abnormal collagen and brittle bones, in the international multicentre phase I/II clinical trial BOOSTB4 (EudraCT: 2015-003669-60, ClinicalTrials.gov ID: NCT03706482).

Fetal MSCs from first trimester tissues have all the typical characteristics of MSCs but are found at a higher frequency, have greater colony-forming capacity and quicker self-renewal cycles [11–13]. Most importantly, fMSCs differentiate more readily into bone and muscle [13–16]. In a study comparing fMSCs from the different first trimester tissues liver, blood, and bone marrow to aMSCs from bone marrow, fMSCs had higher levels of 16 osteogenic genes under basal (non-induced to bone) conditions [16]. Moreover, fMSCs produced more robust osteogenic genes and induced more calcium production in vitro and reached higher levels of osteogenic gene upregulation in vivo and in vitro than adult MSCs under the same conditions [16]. In another study [13], fMSCs were shown to have the highest proliferative and osteogenic potential when comparing four types of MSCs (aMSCs from bone marrow and adipose tissue, MSCs derived from umbilical cord and fMSCs derived from fetal bone marrow) when compared in a direct head-to-head manner.

Several previous attempts to profoundly explain the differences in potentiality described above have been restricted by the then available methods to study gene and protein expression. Two such examples are analyses using gene array expression [17] and RT-PCR [16]. Both these method, and most methods for analysis of proteins are dependent on pre-selected primers/antibodies or target molecules determining what genes or proteins that are possible to study. In the present study we aimed to determine the biosignature at the gene and protein level of fMSCs in comparison to aMSCs and MSC-derived EVs from both sources using RNA Sequencing and Mass Spectrometry. The methods allow for a wide non-biased screening of all genes and proteins expressed by the MSCs and EVs, thereby elucidating the underlying reason for the differences in e.g. the general potentiality and osteogenic capacity between fMSCs and aMSCs demonstrated in vitro, and in animal experiments.

## Methods

### Characterization of MSCs

#### Samples

Fetal MSCs were isolated from donated human fetal liver tissues obtained from legal terminations during the first trimester (embryonic age 5–8 weeks, *n* = 4). For isolation of aMSCs, human bone marrow aspirates were obtained from the iliac crest of healthy donors ranging in age from 6 to 31 years (*n* = 5), see Table 1.

**Table 1:** The donor sex and age of the fetal and adult MSCs.

Donor	Sex	Age at procurement*
Fetal 1	XY	5–5.5 weeks
Fetal 2	XX	7.5 weeks
Fetal 3	Not known	5.5 weeks
Fetal 4	XY	8 weeks
Adult 1	XY	27 years
Adult 2	XY	31 years
Adult 3	XY	30 years
Adult 4	XY	26 years
Adult 5	XX	6 years

*The developmental age of fMSCs is the embryonic age in weeks. XY = male, XX = female

#### Isolation and culture of MSCs

Fetal livers were obtained by dissection and collected in 1 mL of MSC media consisting of Dulbecco’s modified essential medium low glucose, 10% fetal bovine serum, 1% L-glutamine (all Life Technologies, CA, USA), and 1% Antibiotic-Antimycotic (100×, Gibco, Sigma-Aldrich, Missouri, USA), as previously described [18]. The aMSCs were isolated from 10 to 20 mL bone marrow aspirates and expanded as previously described [19], using the same MSC media as used for fMSCs. For the experiments in this study, the fetal and adult MSCs were in Passage (P) 5–8. After isolation, the MSCs were plated at 4000 cells/cm^2^.

#### Surface marker expression

To examine surface markers, the cells were stained with monoclonal antibodies against cluster of differentiation (CD) 73 APC and CD90 FITC (Becton-Dickinson, New Jersey, USA), CD31 PE (Becton-Dickinson), CD45 PerCP (Becton-Dickinson), and human leukocyte antigen (HLA) class II (DR, DP, DQ) PE-Cy7 (Biolegend, San Diego, USA). The antibodies were added to the cells and incubated for 15 min at room temperature in the dark. The cells were washed and then resuspended in Phosphate Buffered Saline (PBS). Finally, the stained cells were analysed in a flow cytometer (FACSVerse, Becton Dickinson). FlowJo (Tree Star version 10.1r5 Inc, Ashland, USA) was used to analyse the data.

#### Cell size

The size of the MSCs was determined by using the CASY TT system (OMNI Life Science OLS, Prague, Czech Republic), that uses a digital pulse processing technique to measure the diameter of cells. The cells were analysed using a 150-µm capillary, and the peak diameters the recorded. The peak diameter is similar to the median diameter, but it is not affected by outliers.

#### Proliferation assays

The proliferation of fetal and adult MSCs was evaluated by consecutive culturing of the cells over time. The number of population doublings, the population doubling time, and the passage at which the cells stopped proliferating was recorded. The cells were kept in culture for up to passage 8 or until senescence. At 70% confluence, the cells were detached using TrypLE (Life Technologies) and viable cells were counted by eosin exclusion in a hemacytometer using 0.01% Eosin (Merck KGaA, Darmstadt, Germany), and replated at the same density (4000 cells/cm^2^) until passage 8. The complete formula of calculation of population doublings and population doubling time is described in detail in Hayflick et al. [20].

#### Senescence assays

The MSCs were stained for β-galactosidase, a hydrolase enzyme that specifically catalyses the hydrolysis of β-galactosidase into monosaccharides in senescent cells. MSCs were analysed for the expression of β-galactosidase using the Senescence Cell Histochemical Staining Kit (Sigma-Aldrich). MSCs at passage 8 were seeded at 4000 cells/cm^2^ in duplicates in 12-well plates (Corning, New York, USA) and kept in culture until 50–70% confluence when the cells were fixed and stained according to the manufacturer’s instructions. The number of cells that stained blue (positive for β-galactosidase), and the total number of cells were counted in five random view fields per well at 10× magnification, and the percentage of β-galactosidase positive cells was calculated.

#### Colony forming unit-fibroblast (CFU-F) assay

A Colony forming units-fibroblast (CFU‐F) assay was performed in triplicates between passage 3–8 by plating 50 cells/well in 6‐well plates (Becton Dickinson, 4 cells/cm^2^) under regular MSC culture conditions. At day 14 the cells were fixed in 100% methanol (Merck KGaA, Darmstadt, Germany) and stained with 0.05% Eosin (Merck KgaA). Colonies consisting of more than 50 cells were counted.

#### Osteoblast differentiation

Osteoblast differentiation was performed using the StemMACS™ OsteoDiff Medium (Miltenyi Biotec, Bergisch Gladbach, Germany). Controls were cultured in MSC media. The MSCs were seeded at 3200 cells/cm^2^ per well in CELLBIND® 12-well plates (Corning) and kept at 37 °C and 5% CO_2_. The medium was replaced every 3–4 days for 16 days. At the end of the experiment, the cells were washed twice with PBS and fixed with 4% formaldehyde (Sigma-Aldrich). Calcium deposition was stained with 2% Alizarin Red S stain (Sigma-Aldrich) at pH 4.2 for 20 min at room temperature under gentle rotation, washed 5 times with dH_2_0, and lastly with PBS to remove unspecific bindings. The cells were viewed using a bright field microscope and images captured at 10× magnification (Olympus, Tokyo, Japan). Cetylpyridinium Chloride (Sigma-Aldrich) was used to elute the dye and the absorbance was measured at 562 nm in an Infinite F200 PRO Tecan spectrophotometer (Tecan, Mannedorf, Switzerland).

#### Adipocyte differentiation

Adipocyte differentiation was performed using the StemMACS™ AdipoDiff Medium (Miltenyi Biotec). Controls were cultured in MSC media. The MSCs were seeded at 21.000 cells/cm^2^ in 12-well plates (Corning) and kept at 37 °C and 5% CO_2_. The medium was replaced every 3–4 days for 21 days. At the end of the experiment, the cells were washed twice with PBS and fixed with 4% formaldehyde (Sigma-Aldrich) for one hour at room temperature. The lipid droplets were stained with 1% Oil Red O (Sigma-Aldrich) for 10 min at room temperature with gentle rotation, then washed 5 times with dH_2_0, and lastly PBS was added to each well. Images were captured using a light inverted microscope (Olympus) at 10× magnification. 100% isopropanol (Sigma-Aldrich) was used to elute the dye and the absorbance was measured at 492 nm in an Infinite F200 PRO Tecan spectrophotometer (Tecan).

### Transcriptomic and proteomic analyses of MSCs & MSC-derived EVs

#### Preparation of MSCs and EVs

MSCs from passage 5–8 were seeded at 4000 cells/cm^2^ and cultured for 7 days in MSC media up to 50–70% confluency, washed twice with PBS, and the media changed to OptiMEM with no serum (Invitrogen, Massachusetts, USA). After two days, the supernatant was collected, centrifuged at 700 *g* for 5 min at room temperature to remove dead cells, and centrifuged again at 2000 *g* for 10 min to remove larger particles and cell debris. The supernatant was filtered through bottle top filters with cellulose acetate membranes with a 0.22 μm pore size (Corning, low protein binding) to remove larger particles. Tangential flow filtration (TFF) was used to isolate the supernatant by diafiltrating with at least two times the initial volume of 0.22 μm filtered PBS and concentrating it to ~ 30 mL using a KR2i TFF system (MicroKross, 20 cm^2^ surface area, SpectrumLabs) equipped with modified polyethersulfone hollow fiber filters with a 300 kDa cut-off. EVs were further purified via bind-elute size exclusion chromatography (BE-SEC): Post concentration by TFF, samples were loaded onto BE-SEC columns (HiScreen Capto Core 700 column, GE Healthcare Life Sciences), connected to an ÄKTAstart chromatography system (GE Healthcare Life Sciences) as described previously [21]. The samples were subsequently filtered through bottle top filters, as described above. Finally, the solution containing the EVs was concentrated to a volume of 500 µL by using an Amicon Ultra-15 10 kDa molecular weight cut-off spin-filter (Merck Millipore). The EV samples from fMSCs and aMSCs were frozen at -70 °C for downstream analyses. For mass spectrometry the EVs were stored in PBS, and for flow cytometry the EVs were stored in PBS-HAT Buffer [22].

Nanoparticle tracking analysis (NTA) was used to assess the EV particle size and concentration. All samples were distinguished by NanoSight NS500 (Malvern, UK) equipped with an NTA 2.3 analytical programme and 488 nm laser. In the light scatter mode, at least five 30 s videos were captured with a camera level of 11–13 frames per sample. Software configurations were kept consistent for all measurements (screen gain 10, detection threshold 7). Before analysis, all samples were filtered through a 0.22 μm filter.

Simultaneously, the fMSCs and aMSCs were harvested using TrypLE (Life Technologies), counted, and aliquots of cell pellets were frozen at -70 °C for RNA and protein isolation (see below).

#### Confirmation of presence of EVs isolated from fMSCs & aMSCs

Multiplex-bead based analysis of surface markers on the EVs was performed by using the human MACSPlex EV kit IO (Miltenyi Biotec). Unless specified, all steps were performed according to an optimized protocol, and as described previously [23]. In brief, EVs were incubated with capture beads (the input dose was 1 × 10^9^ particles as estimated by NTA and diluted to a total volume of 120 µL with PBS), incubated overnight at room temperature for the capture step, and subsequently incubated with a mixture of APC-conjugated pan-tetraspanin antibodies for 1 h followed by washing. The samples were analysed with a MACSQuant Analyzer 10 flow cytometer (Miltenyi Biotec). The data presented are following background subtraction of median APC fluorescence intensity values for each bead population; values obtained for non-EV containing controls (beads + antibodies) were subtracted from sample values (beads + EVs + antibodies) for each bead population. The data were analysed using FlowJo (Tree Star version 10.15 Inc, Ashland, USA).

#### Isolation of RNA and proteins from MSCs & EVs

The frozen MSC and EV pellets were thawed on ice and RNA isolation was performed using the AllPrep DNA/RNA/Protein mini kit (Qiagen, Hilden, Germany) according to the manufacturer’s instructions. The remaining aliquots of MSC and EV pellets were processed to isolate proteins. In the final step, the proteins were dissolved in ALO stabilisation buffer (Qiagen), and frozen at -70 °C for later mass spectrometry analysis.

#### RNA sequencing of MSCs and EVs

Sequencing libraries were prepared according to the Smart-seq2 protocol [24] and cDNA quality was determined on a Bioanalyzer (Agilent Technologies California, USA). Libraries were pooled and 50 bp single-ends were sequenced using Smart-seq2 on Illumina HiSeq3000. Reads in fastq format were aligned to ENSEMBL GRCh37, mapped, and counted in Tophat 2.1.1 to generate a gene count matrix for downstream analysis. After generating a count matrix for detected genes, genes mapping to sex chromosomes were removed to generate more power in downstream differential analysis.

#### Mass Spectrometry analysis of MSCs and EVs

Protein from thawed MSC pellets was purified and resuspended with the AllPrep DNA/RNA/Protein mini kit (Qiagen). Protein resuspensions were frozen at -70 °C before lysis in a lysis buffer (4% SDS, 50 mM HEPES pH 7.6, 1 mM DTT) by broiling at 95 °C and sonication. Lysates were quantified with the DC Protein Assay (Bio-Rad, California, USA), and enriched with a previously described SP3 protein clean up and digestion protocol [25]. Peptides were labelled with TMT10plex (Thermo Scientific, Massachusetts, USA), pooled, and purified with Strata-X SPE (Phenomenex). Pooled proteins were separated using immobilized pH gradient gel strips according to a HiRIEF LC-MS protocol [26]. Peptide fractions were separated with an UltiMate 3000 RSLCnano (Thermo Scientific) and analysed in a Q Exactive HF (Thermo Scientific) mass spectrometer. Peptide identities were inferred from the mass spectrometry spectra via MS-GF + and Percolator as ENSEMBL gene symbols in galaxy [27].

Isolated EVs were analysed by label free quantification. EVs were lysed (2% SDS, 50 mM HEPES pH 7.6, 1 mM DTT) by broiling at 95 °C and sonication, digested with LysC and Trypsin protease (Thermo Scientific), and SP3 enriched as previously described [25]. Peptides were separated on a 5–40% acetonitrile gradient in UltiMate 3000 RSLCnano (Thermo Scientific) and analysed using a Q Exactive HF (Thermo Scientific) mass spectrometer. The sequest percolator node within Proteome Discoverer 1.4 (Thermo Scientific) was used to assign peptides with their UniProt protein IDs at an FDR of < 1%.

#### Bioinformatics

RNA-seq count matrices were filtered for non XY genes and subsetted into protein coding and non-coding data sets via biomaRt. Significantly Differentially Expressed Genes (DEGs) were determined by the False Discovery Rate (FDR) adjusted *P*-value (p-adj) < 0.01 and log2 fold change > 1 after DESeq2 analysis [28]. DESeq2 normalized counts were exported in gct format and collections from Molecular Signature Database were used for GSEA [29, 30]. GSEA results were visualized with bar plots and as enrichment maps with Cytoscape 3.9.1 [31]. Signalling entropy of fetal and adult transcriptomes were assessed in SCENT with the protein-protein interaction matrix [32].

To analyse Differentially Expressed Proteins (DEPs) between fetal and adult MSCs and fetal EVs (fEVs) and adult EVs (aEVs), Differential Expression analysis of quantitative Mass Spectrometry data (DEqMS) was used on label free quantification of EV and HiRIEF peptide-spectrum matches of MSC proteins to determine DEPs [33]. DEPs with FDR adjusted *P*-value < 0.01 and fold change > 2 were used for GO over-representation analysis through ClusterProfiler 4.2.2. DEGs and DEPs were visualized as volcano plots using EnhancedVolcano, and as heatmaps using ComplexHeatmap. DESeq2, DEqMS, and visualization of the results was conducted using R-4.2.2. Venn diagrams were constructed with “VENNY” 2.1, which is an interactive tool for comparing lists with Venn diagrams [34].

### Statistical analysis

Statistical analyses were performed using unpaired two-tailed t-test for comparison of two samples, and using Mann Whitney U-test when the data were not normally distributed, using GraphPad Prism 9.0 (GraphPad software, Inc, La Jolla, CA). All data are shown as mean ± standard deviation. *P*-value < 0.05 was considered statistically significant.

### Data availability

The datasets generated and/or analysed during the current study are not publicly available since the raw RNA-sequencing data can be indirectly traced back to living individuals and are thus considered personal data according to the European General Data Protection Regulation (GDPR). Personal data cannot be shared without the consent of the study participants and unfortunately there is no explicit consent from our study participants for sharing their data.

## Results

### Fetal MSCs are smaller in size and have enhanced capacity for proliferation and colony-formation compared to aMSCs

The fetal and adult MSCs used in this study have previously been fully characterized according to the criteria defined by the International Society for Cellular Therapy [35]. MSCs from four human fetal livers and five adult bone marrow donors were successfully isolated and expanded in vitro. Fetal and adult MSCs expanded between passage 2–8 (fMSCs passage 2–8, aMSCs passage 4–8) consisted of a phenotypically homogeneous cell population when examined by flow cytometric analysis for the expression of surface antigens at passage 5, consistent with published data [18, 19]. Both MSCs sources were negative (< 5%) for CD31, CD45, and HLA class II, and positive (> 95%) for CD73 and CD90 (data not shown).

**Fig. 1:**
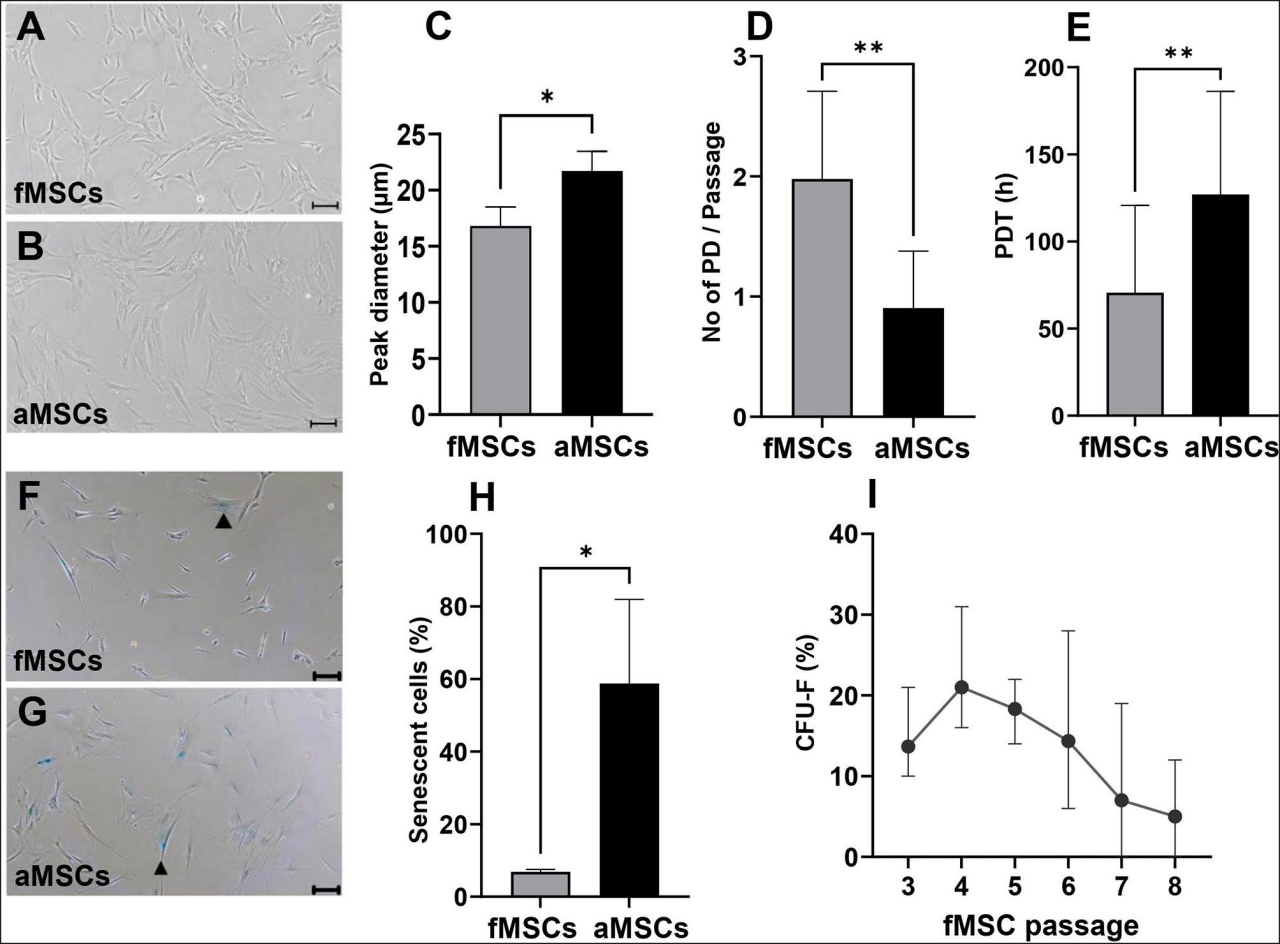
Characteristics of fMSCs and aMSCs. Representative morphologic images of **(A)** fMSCs and **(B)** aMSCs, respectively. **(C)** CASY TT measurement of the mean peak cell diameter of fMSCs and aMSCs (*n* = 3). **(D)** The mean number of population doublings (PD) that fMSCs and aMSCs achieved per passage between passage 5–8, and **(E)** The mean population doubling time (PDT) in hours for fMSCs and aMSCs over passage 5–8. Representative images of β-galactosidase expression in **(F)** fMSCs and **(G)** aMSCs at passage 8. The arrowheads indicate β-galactosidase positive cells (blue), *n* = 3. **(H)** The mean percentage of β-galactosidase positive cells in fMSCs and aMSCs at passage 8 (*n* = 3). **(I)** The mean colony forming unit fibroblast (CFU-F) capacity of fMSCs between passage 3–8, (adult MSCs did not have CFU-F capacity after passage 4), *n* = 3. Mean ± SD, *P < 0.05, **P < 0.01. Scale bars in **(A, B, F, G)** represents 200 μm

Fetal MSCs and aMSCs exhibited a spindle-shaped morphology and adhesion to plastic (Fig. 1A–B). The diameter of fMSCs and aMSCs as measured with CASY TT showed that the mean peak diameter of fMSCs was 16.8 ± 1.87 μm and aMSCs was 21.7 ± 1.6 μm (Fig. 1C), meaning that fMSCs are significantly smaller in size compared to aMSCs (*P* = 0.0257).

Between passage 5 and 8, fMSCs proliferated significantly more than aMSCs and achieved 2.0 ± 0.2 population doublings compared to 0.9 ± 0.2 population doublings per passage (*P* = 0.002, Fig. 1D). The population doubling time was significantly shorter for fMSCs; 70.6 ± 36.8 h compared to 127.0 ± 13.4 for aMSCs per passage (*P* = 0.019, Fig. 1E). Fetal MSCs had significantly fewer β-galactosidase positive (senescent) cells at passage 8 as compared to aMSCs; 6.9 ± 0.6% compared to 58.8 ± 23.2% for aMSCs (*P* = 0.0179, Fig. 1F-H). Lastly, in the CFU-F assay, the fMSCs maintained their colony forming capacity until late passages (passage 7–8) (Fig. 1I), whereas aMSCs did not form colonies after passage 4 (data not shown).

### Fetal MSCs differentiate better into bone than aMSCs

**Fig. 2:**
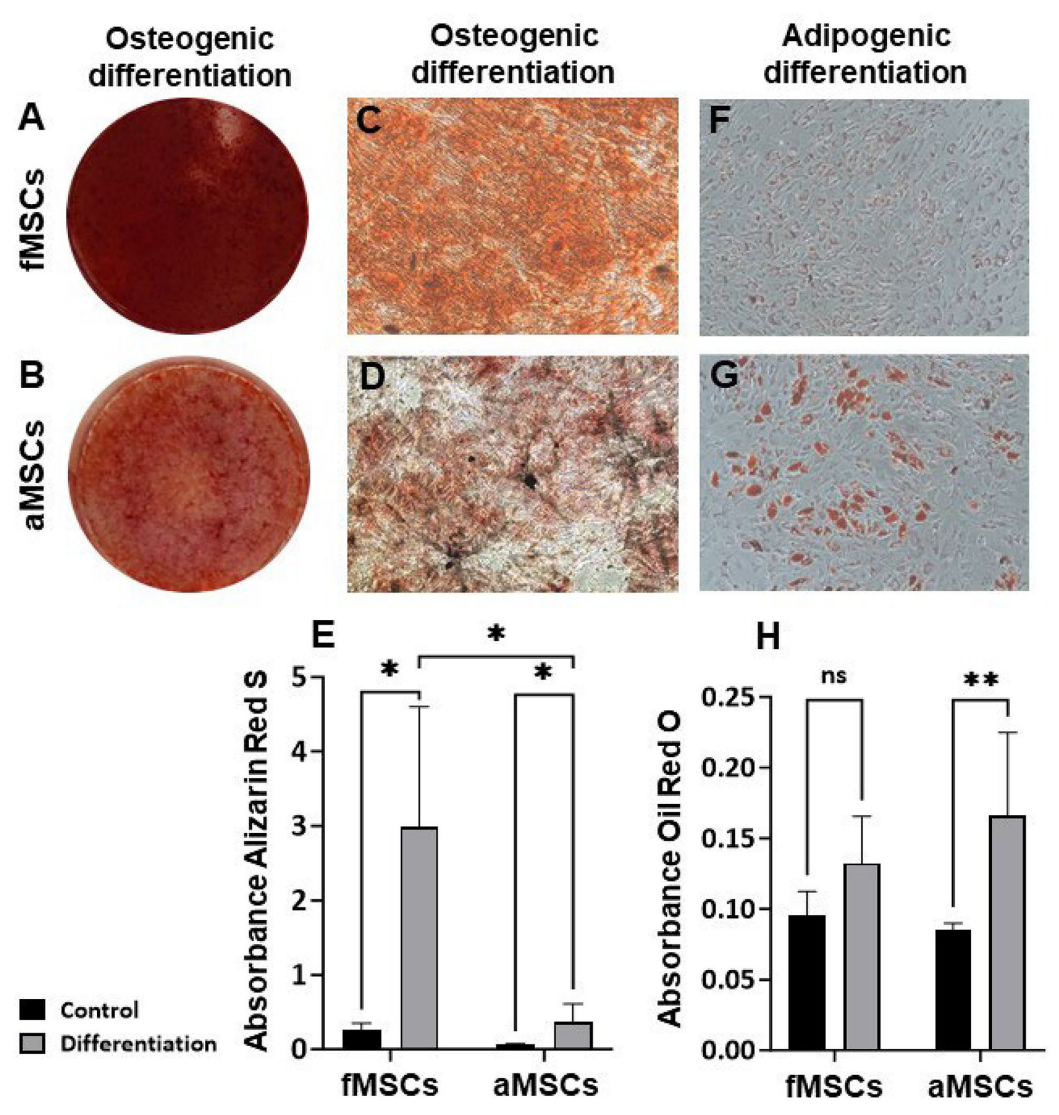
Differentiation of fMSCs and aMSCs into osteoblasts and adipocytes. Representative images of the whole 12-plate wells following Alizarin red S staining of calcium deposits in osteogenic induced MSCs; **(A)** fMSCs and **(B)** aMSCs. Representative microscopic images at 100× magnification of osteogenic induced and Alizarin red S stained (extracellular red staining) **(C)** fMSCs and **(D)** aMSCs. **(E)** Quantification of Alizarin red S staining of osteogenic differentiated and control fMSCs and aMSCs. Representative microscopic images at 100× magnification of **(F)** fMSCs and **(G)** aMSCs induced into adipocytes following Oil Red O staining (red intracellular lipid droplets). **(H)** Quantification of Oil Red O staining of adipogenic differentiated and control fMSCs and aMSCs. Two replicate experiments for each donor, *n* = 3 for fMSCs and aMSCs. Mean ± SD, *P < 0.05, **<0.01, ns = not significant

By culturing MSCs under osteogenic and adipogenic conditions, we investigated the differentiation potential of fMSCs and aMSCs. Both cell types differentiated into both lineages but fMSCs differentiated better into osteoblasts than aMSCs, and aMSCs differentiated better into adipocytes (Fig. 2). Fetal MSCs that had been cultured for more than six passages did not undergo adipogenic differentiation, and aMSCs did not undergo osteogenic differentiation after five passages.

### Fetal and adult MSCs secrete phenotypically similar EVs

**Fig. 3:**
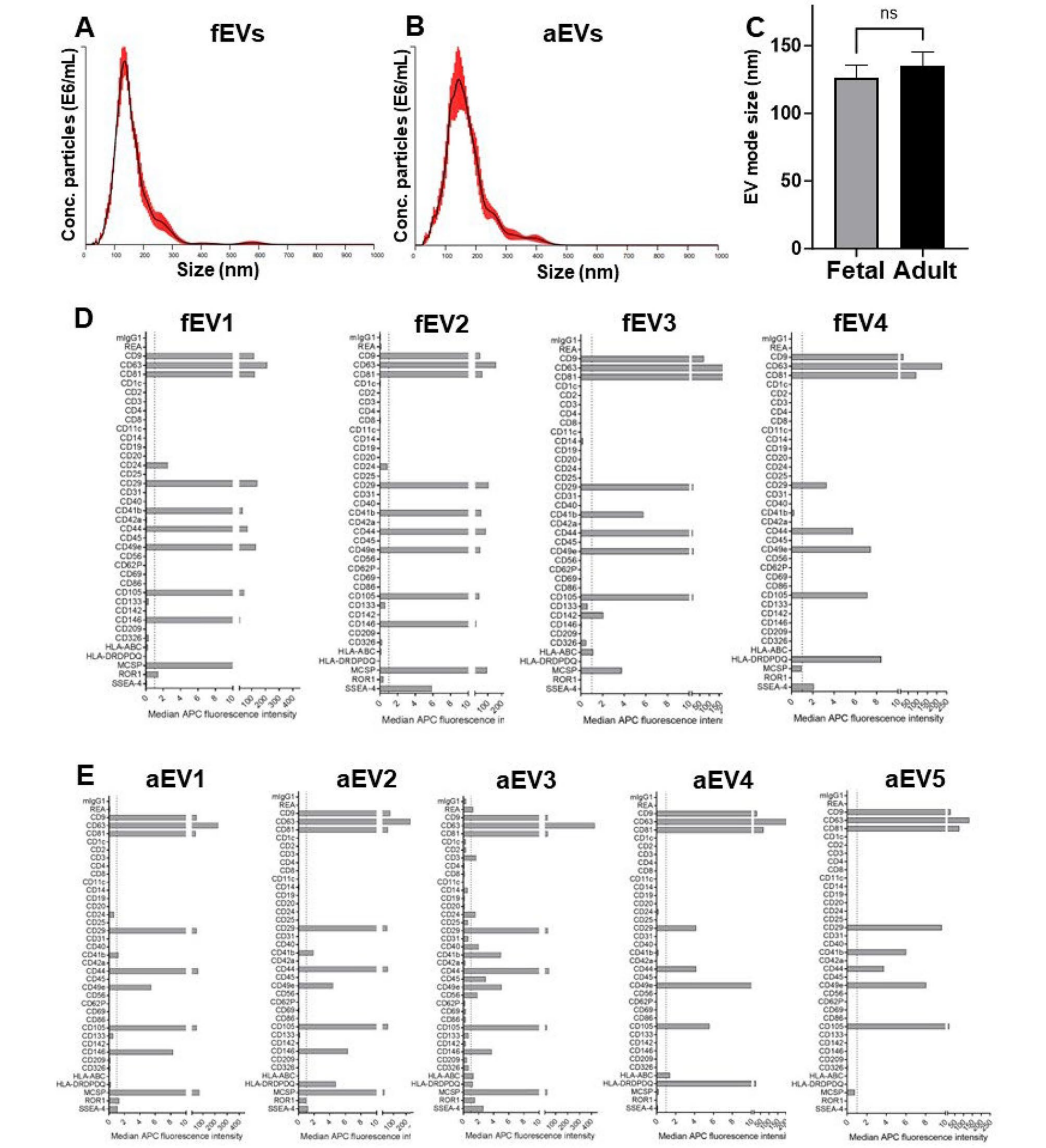
EV concentrations and surface marker expression on the isolated EVs. Nanoparticle tracking analysis (NTA) of extracellular vesicles (EVs), and quantification of proteins on the surface of the EVs by MACSPlex exosome assay. The mode size of **(A)** fetal EVs (fEVs) and **(B)** adult EVs (aEVs), as shown in the representative particle and average size distributions using NTA. The median APC fluorescence intensity values for each protein expressed in the EVs are shown. Averaged Size / Concentration Red lines indicate ± 1 standard deviation of the mean. **(C)** The mean EV mode size distribution of fetal and adult EVs. Surface detection of markers on **(D)** fEVs and **(E)** aEVs. Nine independent experiments were performed from each donor (*n* = 4 biological replicates of fEVs and *n* = 5 biological replicates of aEVs)

EVs were successfully isolated from all fetal and adult MSC cultures. Following isolation of EVs from the supernatant of MSCs using TFF, the EVs were characterized using NTA and multiplex bead-based EV flow cytometry. The mode size of the isolated EVs were similar for fetal and adult EVs (Fig. 3A–B). EV surface marker signature analysis showed the presence of the EV-markers CD9, CD63 and CD81 (Fig. 3C–D), and the MSC markers CD29, CD44, CD49e, CD105 on all fetal and adult EVs. The markers CD41b, CD146, HLA-ABC (HLA class I), HLA DR DP DQ (HLA class II) were detected at different levels for each EV preparation and not on all EV preparations (Fig. 3C–D). No statistically significant differences in average EV size were seen between fEVs, 125.8 ± 9.9 nm (*n* = 4) and aEVs, 135.2 ± 10.1 nm (*n* = 5).

### Fetal & adult MSCs have distinctly different transcriptomic profiles

**Fig. 4:**
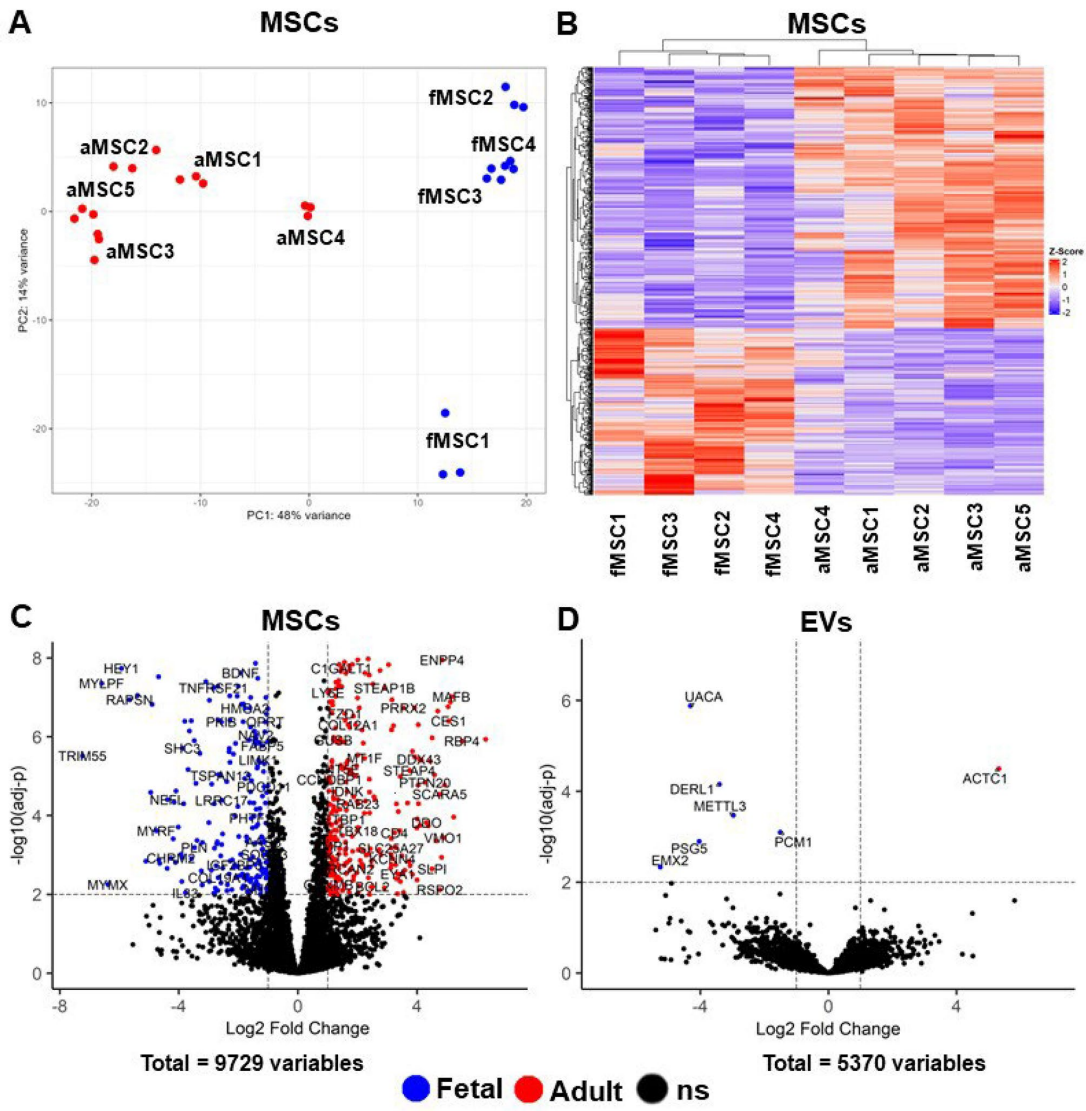
Comparison of the transcriptomes of fetal and adult MSCs and MSC-derived EVs. **(A, B)** Transcriptomics data distribution between fetal and adult MSCs. **(A)** Principal component analysis of fMSCs (blue dots, *n* = 4 donors) and aMSCs (red dots, *n* = 5 donors), (3 replicates from each fMSCs and aMSCs). **(B)** Hierarchal clustering dendrogram of fMSCs and aMSCs illustrating the distinctive donor-type specific transcriptomes. **(C, D)** Comparison of fetal and adult transcriptomes. Volcano plots of **(C)** MSCs and **(D)** EVs. Blue dots show significantly upregulated genes in 4 fMSCs and 3 fEVs and red dots show significantly upregulated genes in 5 aMSCs and aEVs. The coloured dots have adj-*P* < 0.01 and fold change > 2 (|log2 fc|>1). Black dots show detected non-significant (ns) differently expressed genes

To investigate the transcriptomic differences between fetal and adult MSCs and MSC-derived EVs, an analysis of differentially expressed genes (DEG) was performed to identify changes in the gene expression. Principal component analysis (PCA) showed good clustering between donor technical replicates from each MSC (Fig. 4A). The heatmap and PCA plots indicate that fetal and adult MSCs have distinctly transcriptomic profiles (Fig. 4A–B).

After filtering for low count genes, 9729 genes were included in the differential expression analysis, of which 229 genes were significantly upregulated in fMSCs and 359 genes were upregulated in aMSCs (Log2 fold change > 2 and adj-*P* < 0.01) (Fig. 4A). In fMSCs, the most significant genes were linked to muscle contraction pathways; Myogenic Differentiation 1 (*MYOD1*), Tripartite Motif Containing 55 (*TRIM55*), Myosin Light Chain, Phosphorylatable, Fast Skeletal Muscle (*MYLPF*), Fatty Acid Binding Protein 4 (*FABP4*), Myomixer, Myoblast Fusion Factor (*MYMX***)**, whereas for aMSCs the most upregulated genes were Proenkephalin (*PENK*), Cellular Communication Network Factor 5 (*CCN5*), Neurotrophic Receptor Tyrosine Kinase 2 (*NTRK2*), Interleukin 26 (*IL26*), Distal-Less Homeobox 5 (*DLX5*), Keratin 16 (*KRT16*), Lipopolysaccharide Binding Protein (*LBP*), (Family With Sequence Similarity 180 Member A (*FAM180A*) (Log2 fold change > 6).

### Fetal & adult-derived EVs have similar transcriptomic profiles

When comparing fEVs and aEVs, a total of 5379 expressed genes were detected and only six genes were significantly upregulated in fEVs compared to aEVs (fold change > 2 and adj-*P* < 0.01). In fEVs, the significantly upregulated genes were Uveal autoantigen with oiled-coil domains and ankyrin repeats (*UACA*), Derlin 1 (*DERL1*), Methyltransferase 3 (*METTL3*), Pericentriolar Material 1 (*PCM1*), Pregnancy Specific Beta-1-Glycoprotein 5 (*PSG5*), and Empty Spiracles Homeobox 2 (*EMX2*) (Fig. 4D). Only one gene (Actin Alpha Cardiac Muscle 1, *ACTC1*) was significantly upregulated in aEVs compared to fEVs (Fig. 4D).

### Fetal and adult MSCs and EVs co-express several genes

When comparing expressed genes between fMSCs and fEVs, 3098 genes were upregulated in fMSCs and 2363 genes in fEVs (Log2 fold change > 2 and adj-*P* < 0.01) (Supplementary Fig. 1A). Lower numbers of genes were expressed by aMSCs; 1642 genes were upregulated in aMSCs and 1050 genes in aEVs (Log2 fold change > 2 and adj-*P* < 0.01) (Supplementary Fig. 1B). Overlapping and non-overlapping genes were visualized in Venn diagrams (Supplementary Fig. 1C‒E). The most genes were co-expressed by fMSCs and fEVs; 3985 (49.7%), and fMSCs expressed 3197 (39.9%) genes and fEVs expressed 840 (10.5%) genes (Supplementary Fig. 2C). Adult MSCs and EVs co-expressed 3160 (37%) genes, aMSCs 4378 and aEVs 993 (11.6%) genes (Supplementary Fig. 2D). All MSCs and EVs co-expressed 2689 (29.8%) genes (Supplementary Fig. 2E).

### Fetal & adult MSCs are enriched for different pathways

**Fig. 5:**
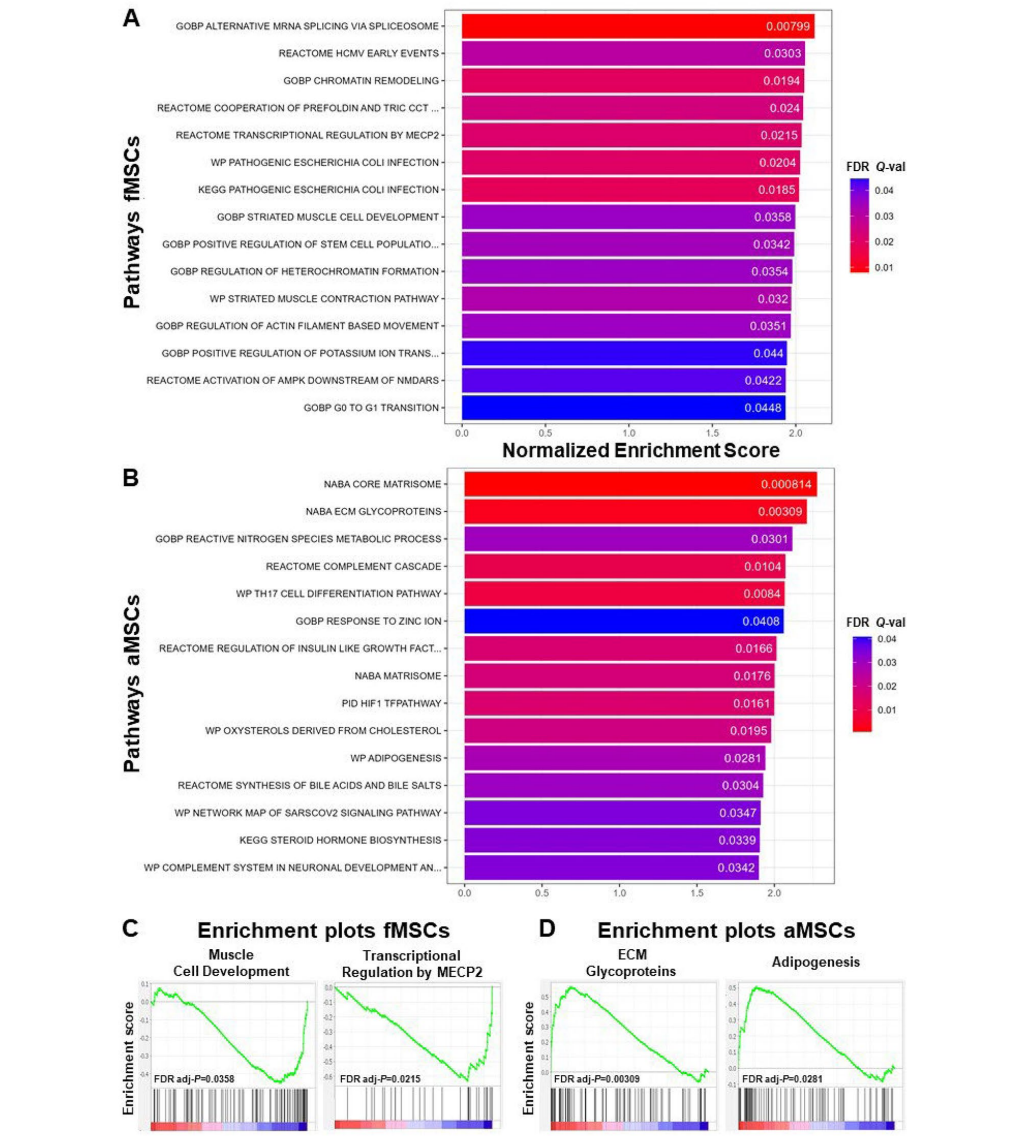
Gene Set Enrichment Analysis (GSEA) comparison of the biological processes’ pathways of fMSCs and aMSCs. **(A, B)** Bar chart of the top 15 significantly enriched pathways in fMSCs **(A)** and aMSCs **(B)**. **(C, D)** Normalized enrichment plots of novel pathways/gene sets enriched in fMSCs (C) and aMSCs **(D)**. The top part of each plot shows the enrichment score that represents running-sum statistic calculated by “walking down” the ranked list of genes. The green line represents the time-course gene expression data (the normalized enrichment score) and the vertical black lines indicate the position of the genes found in the target gene set within a gene list ranked by log2 fold changes. The Enrichment score is the maximum deviation from zero as calculated for each gene going down the ranked list and represents the degree of over-representation of a gene set at the top or the bottom of the ranked gene list. The coloured bar at the bottom of the plot shows positive (red) and negative (blue) correlation to phenotype in fMSCs and aMSCs **(C, D)**

To better understand the biological processes and pathways enriched in fMSCs and aMSCs, we performed Gene Set Enrichment Analysis (GSEA) using GO and KEGG collections obtained from MSigDB. The top 15 significantly upregulated pathways from the GO and KEGG pathway analyses are shown in Fig. 5A–B. Pathways of biological processes significantly enriched in fMSCs were “Positive regulation of stem cell population” (*Q* = 0.034), “Muscle cell development” (*Q* = 0.036), and “Muscle contraction pathway” (*Q* = 0.032). In contrast, biological processes pathways significantly enriched in aMSCs were “Complement cascade” (*Q* = 0.01), “Adipogenesis” (*Q* = 0.028) and “Extracellular matrix glycoproteins” (*Q* = 0.003).

Enrichment plots identified novel gene sets enriched in fMSCs and aMSCs, respectively (Fig. 5C–D). In fMSCs, significantly enriched pathways were “Muscle cell development” (adj-*P*:0.0385, in GOBP) and “Transcriptional regulation by methyl CpG binding protein 2 (MECP2)” (adj-*P*:0.0215, in Reactome), that are associated with metabolic processes and regenerative capacity, in comparison to aMSCs. Conversely, in aMSCs, significantly enrichment pathways were “ECM Glycoproteins” (adj-*P*: 0.00309, in NABA) and “Adipogenesis pathways” (adj-*P*: 0.0281, in WP), when compared to fMSCs (Fig. 5C–D).

**Fig. 6:**
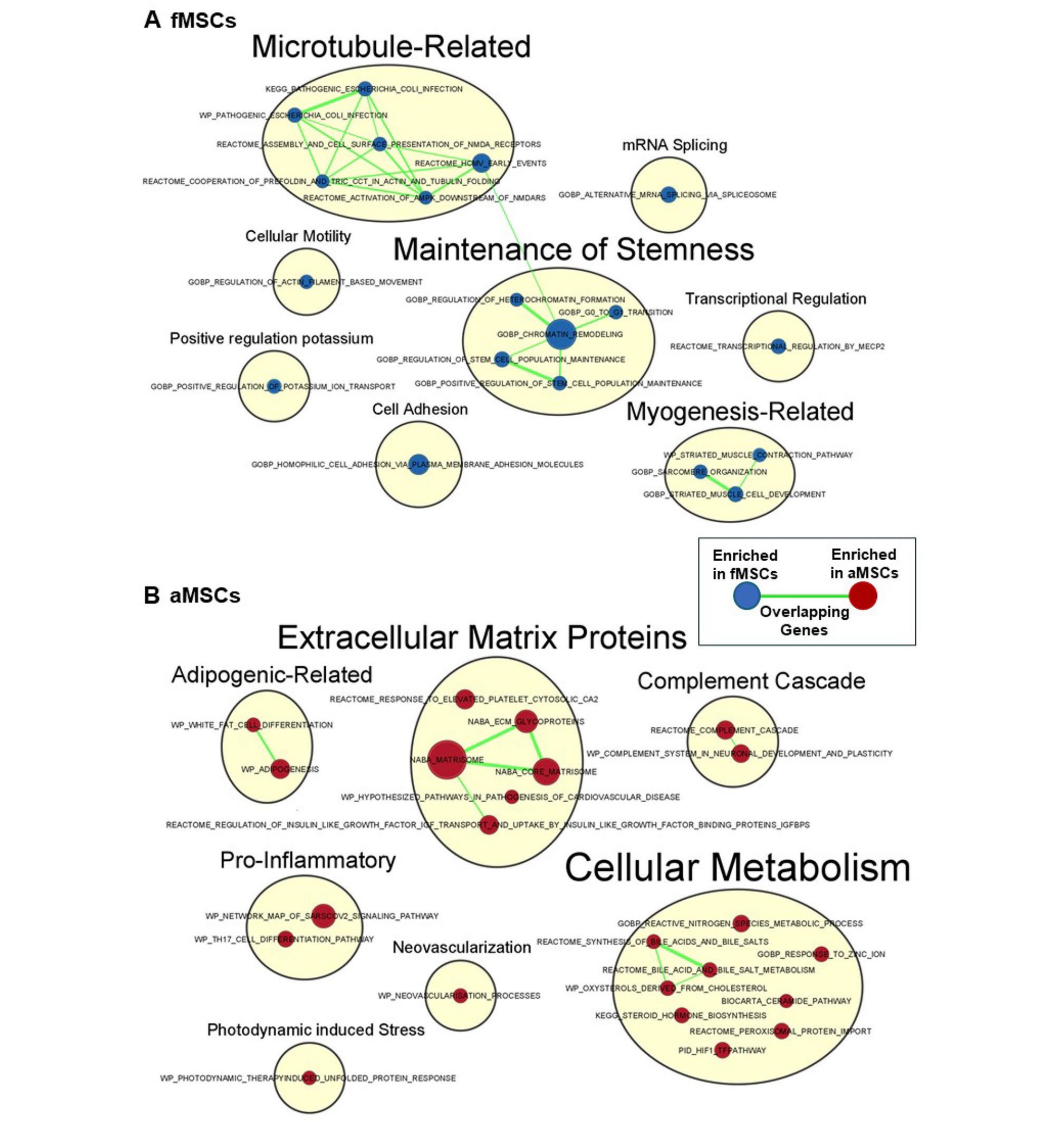
Enrichment maps of transcriptomic differences between fMSCs and aMSCs. Enrichment maps visualizing the gene set enrichment analysis comparing the transcriptomes of **(A)** fMSCs and **(B)** aMSCs using Cytoscape 3.9.1. Individual nodes represent an enriched gene set from C2 or C5 collections with FDR adj-*P* < 0.05. Blue node colour indicates enrichment in fMSCs and red node colour enrichment in aMSCs. The edges of connecting nodes with overlapping genes in different pathways and the thickness of the green lines reflects the magnitude of overlaps. Several nodes were manually clustered into the yellow circles and labelled to describe overarching biological themes

Enrichment maps of GSEA analysis suggest strong enrichment for microtubule related, stemness maintenance and myogenesis related gene sets in fMSCs compared to aMSCs (Fig. 6A). Conversely, aMSCs upregulated gene sets associated with metabolism, extracellular matrix remodelling and adipogenesis (Fig. 6B).

### Fetal MSCs display higher signalling entropy compared to aMSCs

**Fig. 7:**
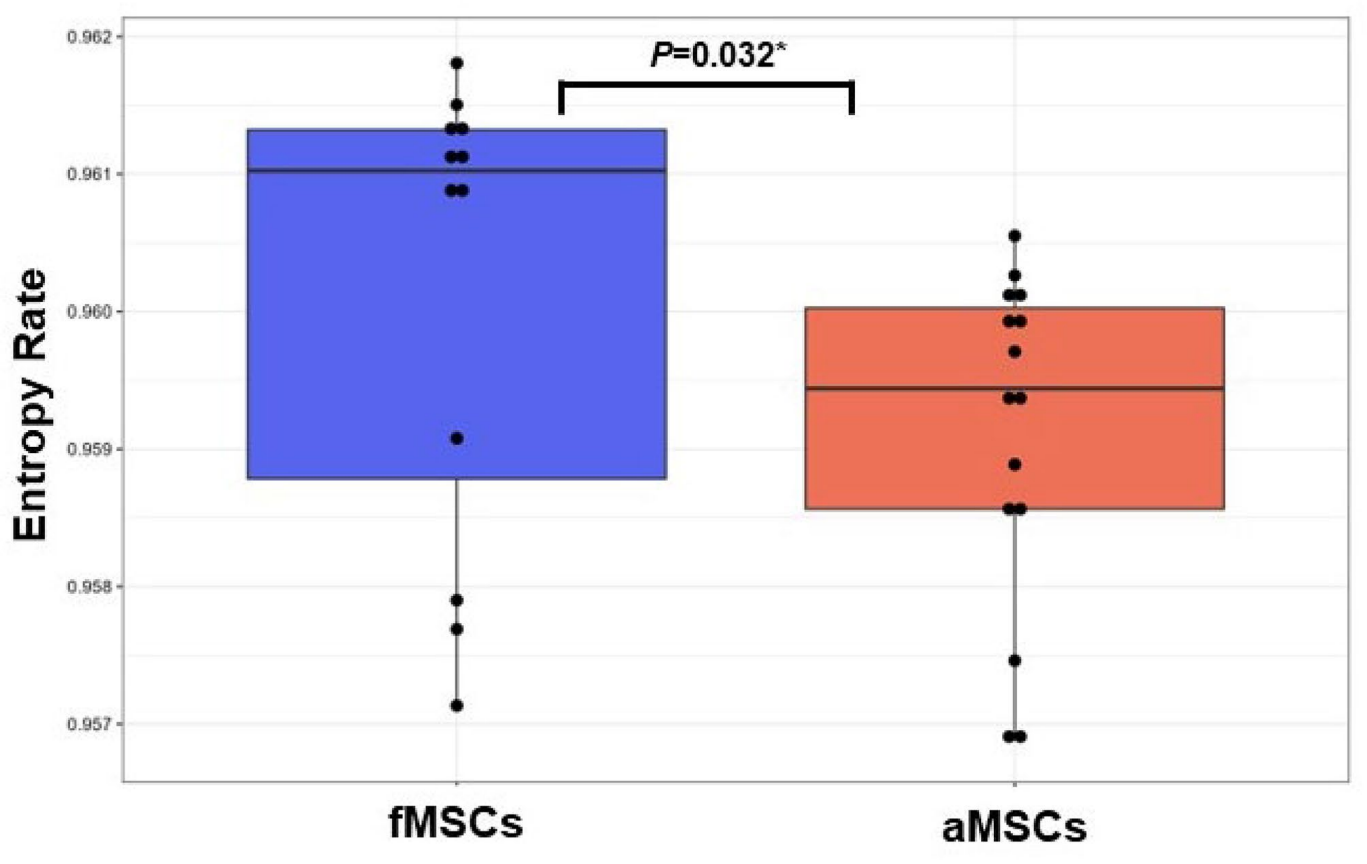
Signalling Entropy analysis of the transcriptomes of fMSCs and aMSCs. Signalling entropy rates obtained in SCENT. Fetal MSCs display statistically significant higher entropy compared to aMSCs (*P* = 0.032). Black nodes represent individual samples including technical replicates of biological samples (fMSCs = 4; aMSCs = 5, *n* = 3 replicates)

Signalling entropy captures the degree of a cell’s theoretical commitment to pre-defined transcriptomic programs using transcriptomic data and a protein connection network. Cells that are less committed to signalling networks have been suggested to have a higher differentiation potential or being in a less differentiated state [36]. In the present study, the transcriptomic data of fMSCs showed significantly higher signalling entropy compared to aMSCs upon examination with a non-parametric two group comparison (*P* = 0.0321, Fig. 7).

### Few proteins are differently expressed between fMSCs & aMSCs and MSC-derived EVs

**Fig. 8:**
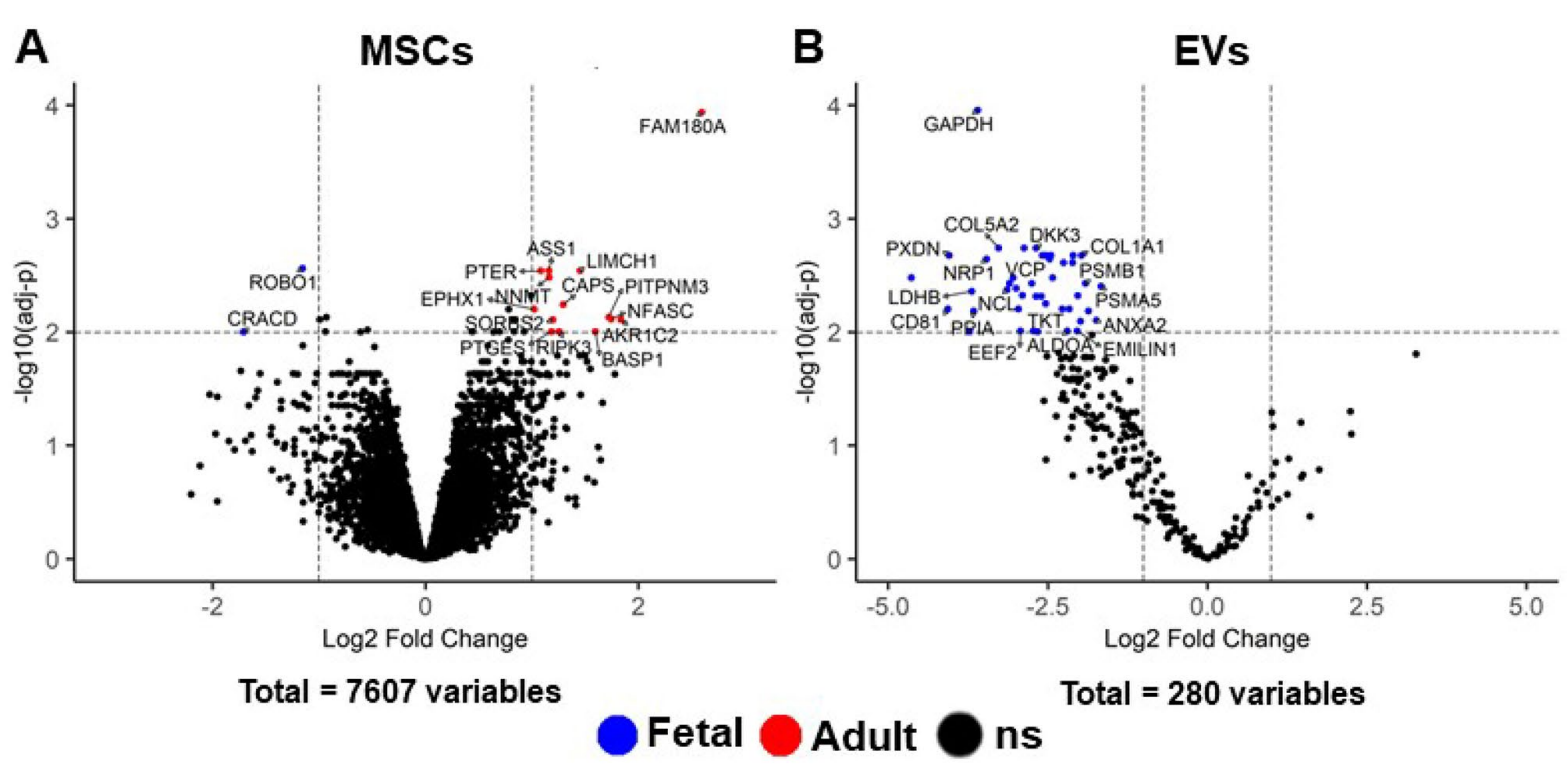
Expressed Proteins in MSCs and MSC-derived EVs. **(A, B)** Volcano plots of proteins in fetal and adult MSCs and MSC-derived EVs analysed by mass spectrometry. The volcano plots show detected proteins (*n* = 7607) in MSCs and EVs (*n* = 280). Blue (fetal) and red (adult) dots show significantly upregulated proteins in **(A)** MSCs (3 fMSCs and 5 aMSCs) and **(B)** EVs (4 fEVs and 5 aEVs). All coloured dots show statistically Differently Expressed Proteins (adj-*P* < 0.01 and |log2 fc| >1) in the MSCs and EVs. The number of proteins detected significantly different in fMSCs = 2 and aMSCs = 14 **(A)**, and in fEVs = 44, none in aEVs **(B)**. Black dots show non-significant (ns) differentially expressed proteins

To identify proteomic differences between fMSCs and aMSCs and MSC-derived EVs, a Differentially Expressed Proteins (DEP) analysis was performed. One of the fMSC donors was discarded due to technical problem (*n* = 3 fetal, *n* = 5 adult). The analysis detected a total of 7607 expressed proteins in all MSCs. Of these proteins, 16 were DEPs (fold change > 2 and adj-*P* < 0.01); 2 proteins were expressed higher in fMSCs compared to aMSCs, and 14 proteins were expressed higher in aMSCs compared to fMSCs, (Fig. 8A and Supplementary Table 1). The two proteins with higher expression in fMSCs compared to aMSCs were Roundabout homolog 1 (ROBO 1), which has a role in cell migration and in the development of the nervous system and other tissues, and Capping protein inhibiting regulator of actin dynamics (CRACD), which is involved in epithelial cell integrity by acting on the maintenance of the actin cytoskeleton. The 14 proteins expressed higher in aMSCs were mainly involved in epithelial cell differentiation pathways; some examples are Phosphotriesterase related (PTER), Brain abundant membrane attached signal protein 1 (BASP1), and Aldo-keto reductase family 1 member C2 (AKR1C2).

A total of 44 proteins were expressed higher in fEVs compared to aEVs, whereas there was no significant difference in protein expression in aEVs compared to fEVs (Fig. 8B and Supplementary Table 1). When the DEPs were investigated in GO terms, the significantly enriched pathways in fEVs were “Collagen fibril organization”, “Protein folding”, “Cellular response to transforming growth factor beta stimulus”, and “Response to transforming growth factor beta” (Table 2). Individual proteins that were found to be significantly enriched in fEVs compared to aEVs included several collagen proteins, and other proteins that have a role in collagen fibril organization, for example, Annexin A2 (ANXA2), Elastin microfibril interfacer 1 (EMILIN1), and Peroxidasin (PXDN). In addition, numerous proteins associated with “Response to transforming growth factor beta” were expressed significantly higher in fEVs compared to aEVs. The full list of pathways and GO terms of the 44 DEP in fEVs are shown in the Supplementary Table 2.

The distribution of proteins was visualized in Venn diagrams (Supplementary Fig. 2A‒C) and shows similar pattern of protein expression in fetal and adult sources. The highest number of proteins were identified in the cells; 5148 (82.4%) and 4138 (79.4%) proteins in fMSCs and aMSCs respectively, and a lesser part were identified in the EVs; 835 (13.4%) and 851 (16.3%) proteins in fEVs and aEVs, respectively. The percentage of proteins that were identified in both MSCs and EVs were the same for fetal and adult sources: 4.3% that equals to 266 proteins in fMSCs and fEVs and 223 proteins in aMSCs and aEVs. As can be seen in Supplementary Fig. 2C, 179 proteins (2.6% of all proteins) were detected in all MSCs and EVs, but all sources of MSCs and EVs also displayed their individual and specific protein profile expression.

### No overlap between expressed genes and proteins in fMSCs versus aMSCs or in fEVs versus aEVs

To detect any overlaps, we compared differently expressed genes and proteins in fMSCs versus aMSCs, and similarly for fEVs versus aEVs. Only one overlap was detected, namely FAM180A (Family with Sequence Similarity 180 Member A) that was expressed higher in aMSCs at both the RNA and protein level compared to fMSCs. The molecular or clinical significance of FAM180A has not been identified.

**Table 2:** Top 10 selected enriched pathways of interest in fEVs compared to aEVs in the gene ontology (GO) analysis (*P*-adj < 0.05)

No	GO Accession ID	GO Name	Gene Symbols	FDR P.adjust
1	GO:0030199	Collagen fibril organization	*COL5A2/PXDN/COL3A1/* *COL1A1/ANXA2/EMILIN1*	5.1E-07
2	GO:0006457	Protein folding	*HSPA8/HSPA5/HSP90AB1/HSP90AA1/QSOX1/PPIA/* *PDIA3/HSPA1A*	5.2E-07
3	GO:0071560	Cellular response to transforming growth factor beta stimulus	*DKK3/COL3A1/COL1A1/* *HSPA5/HSP90AB1/HSPA1A/EMILIN1*	0.2E-4
4	GO:0071559	Response to transforming growth factor beta	*DKK3/COL3A1/COL1A1/* *HSPA5/HSP90AB1/HSPA1A/EMILIN1*	0.2E-4
5	GO:0007179	Transforming growth factor beta receptor signalling pathway	*DKK3/COL3A1/HSPA5/* *HSP90AB1/HSPA1A/* *EMILIN1*	0.5E-4
6	GO:0017015	Regulation of transforming growth factor beta receptor signalling pathway	*DKK3/HSPA5/HSP90AB1/* *HSPA1A/EMILIN1*	0.9E-4
7	GO:1,903,844	Regulation of cellular response to transforming growth factor beta stimulus	*DKK3/HSPA5/HSP90AB1/* *HSPA1A/EMILIN1*	0.9E-4
8	GO:0006757	ATP generation from ADP	*GAPDH/PKM/ENO1/* *ALDOA*	0.3E-3
9	GO:0032388	Positive regulation of intracellular transport	*MSN/YWHAE/HSP90AB1/* *CD81/ANXA2*	0.4E-3
10	GO:0035967	Cellular response to topologically incorrect protein	*HSPA8/VCP/HSPA5/* *HSPA1A*	0.4E-3

## Discussion

Mesenchymal stem/stromal cells (MSCs) are fibroblast-like and spindle-shaped multipotent cells that were first promoted as the future of regenerative medicine due to their differentiation capabilities [9]. However, research has made it clear that MSCs engraft at low levels, but still with a therapeutic effect that often is transient, suggesting that engraftment and differentiation may not be the only mechanism by which MSCs mediate their effects. In fact, findings show that MSCs may also mediate their effects through the release of various factors that influence resident cells via paracrine mechanisms [15, 37–39]. Since the mechanisms are still not fully understood, rigorous characterisation of the cells is an essential step forward in determining the therapeutic effects of MSCs and in developing medicines based on MSCs. In the present study, we therefore performed a detailed characterization and comparison of fetal and adult MSCs phenotypically, and in parallel we determined transcribed genes, proteins, and active pathways and enriched genes and proteins for fMSCs and fEVs in comparison to aMSCs and aEVs. However, to the best of our knowledge, this study is the first that describes fMSCs and fEVs in the transcriptomic and proteomic perspective in comparison to aMSCs and aEVs using wide non-biased RNA Sequencing and Mass spectrometry.

The fetal and adult MSCs and MSC-derived EVs were subjected to identical culture and isolation conditions in the same laboratory to minimize inherent differences. The MSCs have been fully characterized according to the criteria by the International Society for Cellular Therapy [35], and have a fibroblastic morphology, express mesenchymal markers and lack expression of endothelial and hemopoietic markers, and are capable of trilineage differentiation under permissive conditions. We found that fMSCs were significantly smaller in size than aMSCs. It has been shown that there is a relation between the cell size of stem cells and aging, and recently it was reported that biologically older MSCs are larger and have more adenosine triphosphate (ATP) than younger MSCs, and that aged cells also consume ATP at a quicker rate, making them more susceptible to energy shortage [40]. We also found that fMSCs display a less mature state compared to aMSCs and proliferated quicker and achieved more population doublings per passage, had a greater colony-forming capacity and there were fewer senescent cells, which is in line with previous reports [12, 13].

On the transcriptomic level, fetal and adult MSCs displayed good clustering between the donor sources and the technical replicates, but fetal and adult MSCs had distinctly different transcriptomic profiles. In fMSCs, significantly enriched biological processes and pathways comprised of positive regulation of stem cells, muscle cell development, and transcriptional regulation. In the enrichment map for fMSCs, upregulated gene sets included maintenance of stemness, myogenesis, microtubule, and proliferation-related pathways. Gene sets upregulated in aMSCs compared to fMSCs were related to extracellular matrix, the complement cascade and adipogenesis, and in the enrichment maps aMSCs expressed gene sets active in extracellular matrix, cellular metabolism, complement, and adipogenesis pathways, showing a good correlation between the different bioinformatic analyses. Lastly, fMSCs displayed a statistically significant higher signalling entropy of the cell’s undifferentiated state compared to aMSCs. Signalling entropy is a theoretical quantitative, *in silico*, readout of the average undifferentiated state of the profiled cells, recapitulating the known hierarchy of pluripotent, multipotent, and differentiated cell types [36], but does not provide direct evidence of a cell’s true biological regenerative capacity. The data show that as a cell population, fMSCs are positioned higher in the Waddington’s epigenetic landscape compared to aMSCs [41].

It has previously been reported that fMSCs have a greater differentiation potential than aMSCs [14, 17, 42], and differentiate much more readily into bone in vitro and in vivo compared to MSCs derived from umbilical cord, adult bone marrow, or adipose tissue [13, 15, 16]. Similarly, we also observed higher osteogenic differentiation of fMSCs in vitro compared to aMSCs, whereas higher adipogenic differentiation was observed in aMSCs. The higher adipogenic cell differentiation aligns well with the active adipogenesis pathways and gene sets reported in the present study, and with increased gene expression of adipogenic transcripts in previous studies [17, 43]. On the transcriptomic level, fMSCs also showed enrichment of pathways active in muscle development compared to aMSCs, which has previously been documented on the cell level [14]. Interestingly, a study showed that EVs derived from younger rat bone marrow donors had higher expression of the bone markers RUNX2, ALP, and COL1 compared to MSCs obtained from older rats [44]. Taken together, this data indicates that ontogeny appears to be an important determinant for stem cell fitness, which corroborates previous reports [12, 13, 16, 17, 45], meaning that the capacity of the cells and MSC-derived EVs is a function of the biological age.

The proteins detected in the MSCs and EVs in our study largely consisted of typical MSC and EV markers [4, 46], but the fEVs contained significantly more collagen proteins and proteins that have a role in collagen fibril organization, as well as proteins associated with a response to transforming growth factor beta (TGF-β) compared to aEVs. This was also evident when analysing the active pathways, which showed enrichment for collagen and TGF-β in fEVs. Interestingly, in several bone pathologies, including the brittle bone disease Osteogenesis Imperfecta (OI), excessive TGF-β signalling has been reported in bone cells and in the skeleton of mice with OI, and TGF-β neutralizing antibody treatment rescued the bone phenotype in the mice [47–49]. In line with this, a clinical trial reported that the baseline serum from a child with severe OI displayed increased expression and activation of the TGF-β pathway, and that the TGF-β bioactivity was reduced after MSC therapy. This implies that therapy with fMSCs and/or fEVs may positively modulate the TGF-β receptor signalling pathway, and in addition, this therapy may also improve collagen-diseases by the addition of healthy collagen.

A limitation of this study is that the tissues for derivation of the MSCs are of different origin; the fetal liver from the first trimester and bone marrow from the adult. However, they are the two most similar tissues during the respective stages of development. The fetal liver is the blood forming organ during the first trimester and carries out the same functions as the bone marrow in the adult, and the fetal bone marrow does not at that point in development yet possess the same capacity as the adult bone marrow. Hence, the two used tissues are as equivalent as possible.

Allogenic cells are required for treatment of many diseases, and different source materials, like pluripotent cells, fetal or perinatal tissues, or adult bone marrow or adipose can be used as starting material. All source materials have their own inherent advantages and disadvantages, including potentiality/efficacy, need of conditioning regimens, contaminating cells, ease of manufacture and risks for tumours, to mention a few. The use of fetal tissues obtained from elective termination of pregnancy might pose an issue with regards to ethics. To address this, investigations of stakeholder’s views were performed with 56 participants from three groups: adults affected with OI and parents of children affected with OI, health professionals, and patient advocates [50]. There were generally positive views towards using fMSCs as a treatment for OI. A clinical trial, BOOSTB4, is currently performed where it is investigated if the highly bone-forming fMSCs is a safe and efficient treatment of severe types of OI.

## Conclusion

The key finding from this study is that fMSCs are in a more undifferentiated state at the transcriptomic, proteomic, and cellular levels, and that they possess a greater bone formation and regenerative capacity compared to aMSCs. We also show that the active pathways in fetal and adult MSCs are distinctly different, mirroring their developmental origin and potential in regenerative medicine.

### Electronic supplementary material

Below is the link to the electronic supplementary material.

Supplementary Material 1

## Data Availability

The datasets generated and/or analysed during the current study are not publicly available simce the raw RNA-sequencing data can be indirectly traced back to living individuals and are thus considered personal data according to the European General Data Protection Regulation (GDPR). Personal data cannot be shared without the consent of the study participants and unfortunately there is no explicit consent from our study participants for sharing their data.

## Abbreviations

aEVs: adult extracellular vesicles
aMSCs: adult mesenchymal stem/stromal cells
CD: cluster of differentiation
CFU-F: colony-forming unit-fibroblast
DEG: differentially expressed genes
DEP: differentially expressed proteins
EVs: extracellular vesicles
fEVs: fetal extracellular vesicles
fMSCs: fetal mesenchymal stem/stromal cells
GO: gene ontology
GSEA: gene set enrichment analysis
HLA: human leukocyte antigen
KEGG: Kyoto encyclopedia of genes and genomes
MSCs: mesenchymal stem/stromal cells
PBS: phosphate buffered saline
PCA: principal component analysis
PD: population doublings
PDT: population doubling time
RNA: ribonucleic acid
SCENT: signalling cell entropy
TFF: tangential flow filtration
