# Supplementary material for: Transcriptomic and proteomic profiles of fetal versus adult mesenchymal stromal cells and mesenchymal stromal cell-derived extracellular vesicles
Source: Stem Cell Res Ther. 2024 Mar 13;15:77. doi: 10.1186/s13287-024-03683-7 (PMC10935839; doi:10.1186/s13287-024-03683-7)
Supplement: Supplementary file 1 — Supplementary Material 1 [file 13287_2024_3683_MOESM1_ESM.docx]

**Supplementary Table 1.** Differentially expressed proteins (DEPs) in adult and fetal MSCs and fetal EVs (log 2 fc>1, adj-*P*<0.01).

| **Upregulated in** | **Protein** | **UniProtKB** | **logFC** | **sca.adj.pval**  **(DEqMS *P*-values)** |
| --- | --- | --- | --- | --- |
| **fMSCs**  2 proteins | ROBO1 | Q2M1J3 | -1.15467 | 0.002731 |
|  | CRACD | Q6ZU35 | -1.71032 | 0.009921 |
| **aMSCs**  14 proteins | FAM180A | Q6UWF9 | 2.598006 | 0.000115 |
|  | PTER | Q96BW5 | 1.085607 | 0.002878 |
|  | LIMCH1 | Q9UPQ0 | 1.451999 | 0.002878 |
|  | ASS1 | P00966 | 1.160675 | 0.002878 |
|  | NNMT | P40261 | 1.162408 | 0.003291 |
|  | CAPS | Q9ULU8 | 1.292346 | 0.00572 |
|  | EPHX1 | P07099 | 1.021451 | 0.006296 |
|  | PITPNM3 | Q9BZ71 | 1.722478 | 0.007386 |
|  | NFASC | O94856 | 1.746567 | 0.007643 |
|  | AKR1C2 | P52895 | 1.836126 | 0.007762 |
|  | SORBS2 | O94875 | 1.196977 | 0.007762 |
|  | PTGES | O14684 | 1.183923 | 0.009882 |
|  | RIPK3 | Q9Y572 | 1.256599 | 0.009882 |
|  | BASP1 | P80723 | 1.597315 | 0.009882 |
| **fEVs**  44 proteins | GAPDH | P04406 | -3.59777 | 0.00011 |
|  | HSPA8 | P11142 | -2.87113 | 0.001809 |
|  | COL5A2 | P05997 | -3.27359 | 0.001809 |
|  | DKK3 | Q9UBP4 | -2.68351 | 0.001809 |
|  | PXDN | Q92626 | -4.04337 | 0.002102 |
|  | COL3A1 | P02461 | -2.53087 | 0.002102 |
|  | NAW | P02458-1 | -2.58764 | 0.002102 |
|  | PKM | P14618 | -2.44933 | 0.002102 |
|  | NA | P14618-2 | -2.44933 | 0.002102 |
|  | COL1A1 | P02452 | -1.97557 | 0.002102 |
|  | NA | P05067-4 | -2.10315 | 0.002102 |
|  | NRP1 | O14786 | -3.46143 | 0.002268 |
|  | PCOLCE | Q15113 | -2.47317 | 0.002268 |
|  | PSMA1 | P25786 | -2.11116 | 0.002435 |
|  | MSN | P26038 | -2.25132 | 0.002435 |
|  | NA | Q14112-2 | -4.63604 | 0.003307 |
|  | VCP | P55072 | -3.04391 | 0.003307 |
|  | YWHAE | P62258 | -2.42308 | 0.003307 |
|  | PSMB1 | P20618 | -1.91299 | 0.003722 |
|  | YWHAZ | P63104 | -2.75298 | 0.003722 |
|  | NA | P14543-2 | -3.09954 | 0.003722 |
|  | PSMA5 | P28066 | -1.66634 | 0.003952 |
|  | NA | P22392-2 | -2.99439 | 0.004103 |
|  | NCL | P19338 | -3.13527 | 0.004215 |
|  | LDHB | P07195 | -3.69284 | 0.004368 |
|  | CFL1 | P23528 | -2.89681 | 0.00473 |
|  | HSPA5 | P11021 | -2.0283 | 0.004766 |
|  | HSP90AB1 | P08238 | -2.68452 | 0.004834 |
|  | YWHAG | P61981 | -2.60204 | 0.004834 |
|  | HSP90AA1 | P07900 | -2.53247 | 0.005597 |
|  | ENO1 | P06733 | -2.1668 | 0.006225 |
|  | CD81 | P60033 | -4.06858 | 0.006225 |
|  | NA | Q14315-2 | -2.95835 | 0.006225 |
|  | QSOX1 | O00391 | -2.27193 | 0.006225 |
|  | PPIA | P62937 | -3.66361 | 0.006486 |
|  | PDIA3 | P30101 | -1.86159 | 0.006486 |
|  | ANXA2 | P07355 | -1.7401 | 0.007845 |
|  | HSPA1B/HSPA1A | P0DMV8 | -1.98891 | 0.007994 |
|  | ALDOA | P04075 | -2.19508 | 0.009748 |
|  | EMILIN1 | Q9Y6C2 | -2.04195 | 0.009748 |
|  | TKT | P29401 | -2.72584 | 0.009748 |
|  | EEF2 | P13639 | -2.928 | 0.009748 |
|  | NA | P60174-1 | -2.66088 | 0.009854 |
|  | NA | P10909-4 | -3.72161 | 0.009854 |

NA=not applicable.

**Supplementary Table 2.** Full list of enriched pathways in fEVs compared to aEVs proteins in the gene ontology analysis (*P*-adj<0.05).

| **ID** | **Description** | **Protein ID** | ***P*-value** | ***P*.adjust** | ***Q*-value** | **Count*** |
| --- | --- | --- | --- | --- | --- | --- |
| GO:0030199 | Collagen fibril organization | COL5A2/PXDN/COL3A1/COL1A1/ANXA2/EMILIN1 | 3.73E-09 | 5.14E-06 | 3.23E-06 | 6 |
| GO:0006457 | Protein folding | HSPA8/HSPA5/HSP90AB1/HSP90AA1/QSOX1/PPIA/PDIA3/HSPA1A | 7.55E-09 | 5.20E-06 | 3.27E-06 | 8 |
| GO:0042026 | Protein refolding | HSPA8/HSPA5/HSP90AA1/HSPA1A | 2.50E-07 | 0.000115 | 7.21E-05 | 4 |
| GO:0034605 | Cellular response to heat | VCP/YWHAE/HSP90AB1/HSP90AA1/HSPA1A | 3.33E-07 | 0.000115 | 7.21E-05 | 5 |
| GO:0006986 | Response to unfolded protein | HSPA8/VCP/HSPA5/HSP90AB1/HSP90AA1/HSPA1A | 4.38E-07 | 0.000121 | 7.59E-05 | 6 |
| GO:0071560 | Cellular response to transforming growth factor beta stimulus | DKK3/COL3A1/COL1A1/HSPA5/HSP90AB1/HSPA1A/EMILIN1 | 8.95E-07 | 0.000204 | 0.000128 | 7 |
| GO:0071559 | Response to transforming growth factor beta | DKK3/COL3A1/COL1A1/HSPA5/HSP90AB1/HSPA1A/EMILIN1 | 1.04E-06 | 0.000204 | 0.000128 | 7 |
| GO:0035966 | Response to topologically incorrect protein | HSPA8/VCP/HSPA5/HSP90AB1/HSP90AA1/HSPA1A | 1.22E-06 | 0.00021 | 0.000132 | 6 |
| GO:1903362 | Regulation of cellular protein catabolic process | MSN/VCP/HSP90AB1/HSP90AA1/CD81/ANXA2/HSPA1A | 1.65E-06 | 0.000252 | 0.000158 | 7 |
| GO:0007179 | Transforming growth factor beta receptor signaling pathway | DKK3/COL3A1/HSPA5/HSP90AB1/HSPA1A/EMILIN1 | 3.41E-06 | 0.00047 | 0.000296 | 6 |
| GO:0009408 | Response to heat | VCP/YWHAE/HSP90AB1/HSP90AA1/HSPA1A | 5.15E-06 | 0.000645 | 0.000406 | 5 |
| GO:0017015 | Regulation of transforming growth factor beta receptor signaling pathway | DKK3/HSPA5/HSP90AB1/HSPA1A/EMILIN1 | 7.04E-06 | 0.000807 | 0.000508 | 5 |
| GO:1903844 | Regulation of cellular response to transforming growth factor beta stimulus | DKK3/HSPA5/HSP90AB1/HSPA1A/EMILIN1 | 8.87E-06 | 0.000939 | 0.000591 | 5 |
| GO:1903320 | Regulation of protein modification by small protein conjugation or removal | VCP/HSPA5/HSP90AB1/HSP90AA1/PPIA/HSPA1A | 1.30E-05 | 0.001274 | 0.000802 | 6 |
| GO:0051131 | Chaperone-mediated protein complex assembly | HSP90AB1/HSP90AA1/HSPA1A | 1.89E-05 | 0.001737 | 0.001093 | 3 |
| GO:1903364 | Positive regulation of cellular protein catabolic process | MSN/VCP/HSP90AA1/CD81/HSPA1A | 2.64E-05 | 0.002269 | 0.001428 | 5 |
| GO:0008037 | Cell recognition | NRP1/MSN/YWHAZ/CD81/ALDOA | 3.66E-05 | 0.002763 | 0.001738 | 5 |
| GO:0006096 | Glycolytic process | GAPDH/PKM/ENO1/ALDOA | 3.72E-05 | 0.002763 | 0.001738 | 4 |
| GO:0006757 | ATP generation from ADP | GAPDH/PKM/ENO1/ALDOA | 3.81E-05 | 0.002763 | 0.001738 | 4 |
| GO:0034620 | Cellular response to unfolded protein | HSPA8/VCP/HSPA5/HSPA1A | 4.62E-05 | 0.003104 | 0.001953 | 4 |
| GO:0051085 | Chaperone cofactor-dependent protein refolding | HSPA8/HSPA5/HSPA1A | 4.92E-05 | 0.003104 | 0.001953 | 3 |
| GO:0009266 | Response to temperature stimulus | VCP/YWHAE/HSP90AB1/HSP90AA1/HSPA1A | 4.96E-05 | 0.003104 | 0.001953 | 5 |
| GO:0046031 | ADP metabolic process | GAPDH/PKM/ENO1/ALDOA | 6.20E-05 | 0.003711 | 0.002334 | 4 |
| GO:0033120 | Positive regulation of RNA splicing | HSPA8/NCL/HSPA1A | 6.88E-05 | 0.00395 | 0.002485 | 3 |
| GO:0006165 | Nucleoside diphosphate phosphorylation | GAPDH/PKM/ENO1/ALDOA | 7.34E-05 | 0.004044 | 0.002545 | 4 |
| GO:0046939 | Nucleotide phosphorylation | GAPDH/PKM/ENO1/ALDOA | 7.97E-05 | 0.004083 | 0.002569 | 4 |
| GO:0051084 | 'De novo' posttranslational protein folding | HSPA8/HSPA5/HSPA1A | 8.21E-05 | 0.004083 | 0.002569 | 3 |
| GO:0031396 | Regulation of protein ubiquitination | HSPA5/HSP90AB1/HSP90AA1/PPIA/HSPA1A | 8.30E-05 | 0.004083 | 0.002569 | 5 |
| GO:0006090 | Pyruvate metabolic process | GAPDH/PKM/ENO1/ALDOA | 9.90E-05 | 0.00434 | 0.00273 | 4 |
| GO:0032388 | Positive regulation of intracellular transport | MSN/YWHAE/HSP90AB1/CD81/ANXA2 | 9.97E-05 | 0.00434 | 0.00273 | 5 |
| GO:0009135 | Purine nucleoside diphosphate metabolic process | GAPDH/PKM/ENO1/ALDOA | 0.000101 | 0.00434 | 0.00273 | 4 |
| GO:0009179 | Purine ribonucleoside diphosphate metabolic process | GAPDH/PKM/ENO1/ALDOA | 0.000101 | 0.00434 | 0.00273 | 4 |
| GO:1902949 | Positive regulation of tau-protein kinase activity | HSP90AB1/HSP90AA1 | 0.000108 | 0.004396 | 0.002766 | 2 |
| GO:0009185 | Ribonucleoside diphosphate metabolic process | GAPDH/PKM/ENO1/ALDOA | 0.000109 | 0.004396 | 0.002766 | 4 |
| GO:0006458 | 'De novo' protein folding | HSPA8/HSPA5/HSPA1A | 0.000113 | 0.004396 | 0.002766 | 3 |
| GO:0035967 | Cellular response to topologically incorrect protein | HSPA8/VCP/HSPA5/HSPA1A | 0.000115 | 0.004396 | 0.002766 | 4 |
| GO:1905323 | Telomerase holoenzyme complex assembly | HSP90AB1/HSP90AA1 | 0.000146 | 0.005417 | 0.003408 | 2 |
| GO:0045732 | Positive regulation of protein catabolic process | MSN/VCP/HSP90AA1/CD81/HSPA1A | 0.000157 | 0.005689 | 0.003579 | 5 |
| GO:2000767 | Positive regulation of cytoplasmic translation | PKM/EEF2 | 0.000166 | 0.005869 | 0.003692 | 2 |
| GO:0009132 | Nucleoside diphosphate metabolic process | GAPDH/PKM/ENO1/ALDOA | 0.000186 | 0.006077 | 0.003824 | 4 |
| GO:0034975 | Protein folding in endoplasmic reticulum | HSPA5/PDIA3 | 0.000188 | 0.006077 | 0.003824 | 2 |
| GO:1900122 | Positive regulation of receptor binding | NRP1/ANXA2 | 0.000188 | 0.006077 | 0.003824 | 2 |
| GO:0090092 | Regulation of transmembrane receptor protein serine/threonine kinase signaling pathway | DKK3/HSPA5/HSP90AB1/HSPA1A/EMILIN1 | 0.00019 | 0.006077 | 0.003824 | 5 |
| GO:0006754 | ATP biosynthetic process | VCP/ENO1/ALDOA | 0.000203 | 0.006301 | 0.003964 | 3 |
| GO:0034976 | Response to endoplasmic reticulum stress | VCP/HSPA5/PDIA3/HSPA1A/EEF2 | 0.000206 | 0.006301 | 0.003964 | 5 |
| GO:1902947 | Regulation of tau-protein kinase activity | HSP90AB1/HSP90AA1 | 0.000262 | 0.007854 | 0.004941 | 2 |
| GO:0085029 | Extracellular matrix assembly | PXDN/QSOX1/EMILIN1 | 0.000273 | 0.008002 | 0.005034 | 3 |
| GO:0019062 | Virion attachment to host cell | HSP90AB1/CD81 | 0.000319 | 0.009037 | 0.005686 | 2 |
| GO:0061077 | Chaperone-mediated protein folding | HSPA8/HSPA5/HSPA1A | 0.000322 | 0.009037 | 0.005686 | 3 |
| GO:0016052 | Carbohydrate catabolic process | GAPDH/PKM/ENO1/ALDOA | 0.000345 | 0.009231 | 0.005808 | 4 |
| GO:0009206 | Purine ribonucleoside triphosphate biosynthetic process | VCP/ENO1/ALDOA | 0.000348 | 0.009231 | 0.005808 | 3 |
| GO:1902946 | Protein localization to early endosome | NRP1/MSN | 0.000349 | 0.009231 | 0.005808 | 2 |
| GO:0009145 | Purine nucleoside triphosphate biosynthetic process | VCP/ENO1/ALDOA | 0.000366 | 0.009508 | 0.005982 | 3 |
| GO:0044650 | Adhesion of symbiont to host cell | HSP90AB1/CD81 | 0.00038 | 0.009514 | 0.005985 | 2 |
| GO:0044794 | Positive regulation by host of viral process | HSPA8/CFL1 | 0.00038 | 0.009514 | 0.005985 | 2 |

*Count= Number of proteins in the pathway.

**
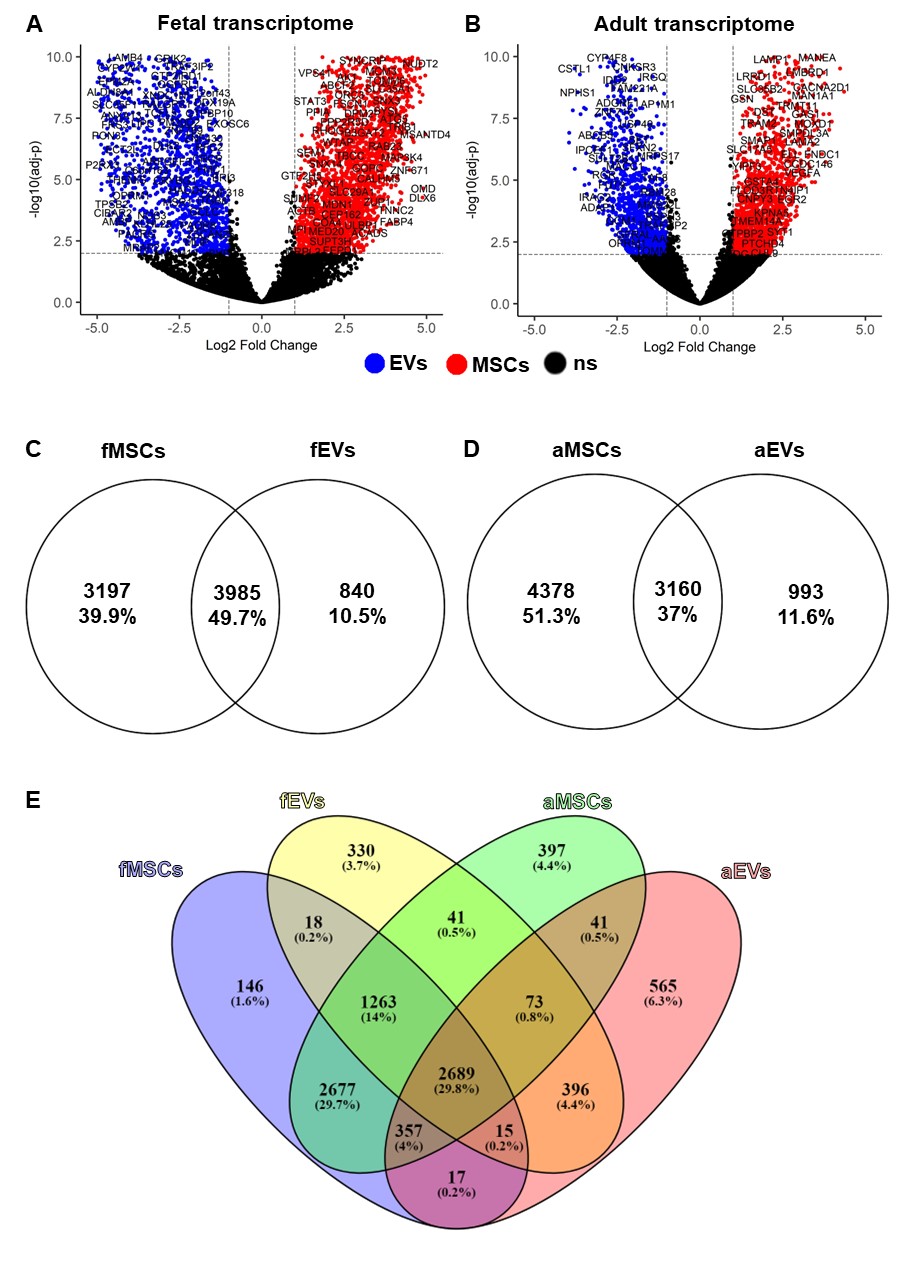
**

***Supplementary Figure 1. Number of overlapping and non-overlapping genes in MSCs and MSC-derived EVs.***

***(A, B)*** *Comparison* *of fetal and adult MSC and EV transcriptomes. Volcano plots of* ***(A)*** *fMSCs and fEVs and* ***(B)*** *aMSCs and aEVs. Blue dots show significantly upregulated genes in 4 fMSCs and 5 aMSCs and red dots show significantly upregulated genes in and 3 fEVs and 5 aEVs. The coloured dots have adj-P<0.01 and fold change>2 (|log2 fc|>1). Black dots show detected non-significant (ns) differently expressed genes.* ***(C‒E)*** *Venn diagrams showing the distribution of all expressed genes detected in each MSC and EV type (total number and percentage with no threshold applied) in* ***(C)*** *fMSCs and fEVs,* ***(D)*** *aMSCs and aEVs, and (E) between all the investigated MSCs and EVs (fMSCs, fEVs, aMSCs, aEVs).*


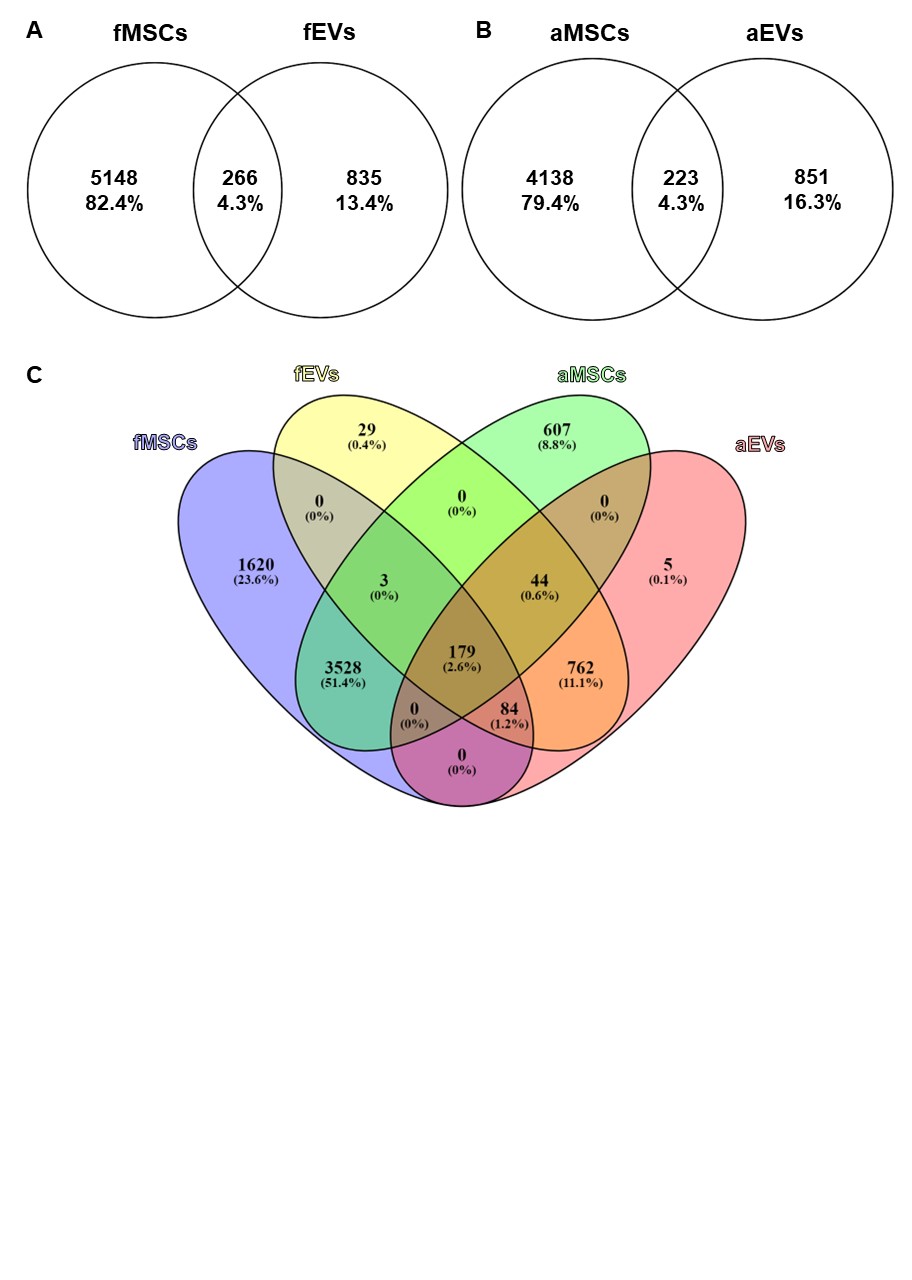


***Supplementary Figure 2. Distribution of expressed proteins in MSCs and MSC-derived EV*s.**

*Venn diagrams showing the protein expression (number of detected proteins and the percentage per MSC and EV type with no threshold applied) distributed between shared and non-shared proteins in* ***(A)*** *fMSCs and fEVs,* ***(B)*** *aMSCs and aEVs, and* ***(C)*** *between the investigated MSCs and EVs (fMSCs, fEVs, aMSCs, aEVs).*
